# The ATM Kinase Inhibitor AZD0156 Is a Potent Inhibitor of *Plasmodium* Phosphatidylinositol 4‐Kinase (PI4Kβ) and Is an Attractive Candidate for Medicinal Chemistry Optimization Against Malaria

**DOI:** 10.1002/anie.202425206

**Published:** 2025-05-15

**Authors:** John G. Woodland, Dina Coertzen, Kathryn J. Wicht, Virginia Franco Hidalgo, Charisse Flerida A. Pasaje, Luiz C. Godoy, Tarrick Qahash, Mmakwena M. Mmonwa, Godwin A. Dziwornu, Lynn Wambua, Sarah Harries, Constance M. Korkor, Mathew Njoroge, Liezl Krugmann, Dale Taylor, Meta Leshabane, Henrico Langeveld, Tayla Rabie, Janette Reader, Mariëtte van der Watt, Nelius Venter, Erica Erlank, Ayesha S. Aswat, Lizette L. Koekemoer, Tomas Yeo, Jin H. Jeon, David A. Fidock, Francisco Javier Gamo, Sergio Wittlin, Jacquin C. Niles, Manuel Llinas, Lauren B. Coulson, Lyn‐Marié Birkholtz, Kelly Chibale

**Affiliations:** ^1^ Holistic Drug Discovery and Development (H3D) Centre University of Cape Town Rondebosch Cape Town 7701 South Africa; ^2^ South African Medical Research Council Drug Discovery and Development Research Unit Institute of Infectious Disease and Molecular Medicine University of Cape Town Cape Town 7925 South Africa; ^3^ Department of Chemistry University of Cape Town Rondebosch Cape Town 7701 South Africa; ^4^ Department of Biochemistry, Genetics and Microbiology Institute for Sustainable Malaria Control University of Pretoria Hatfield 0028 South Africa; ^5^ GlaxoSmithKline Tres Cantos Medicines Development Campus Madrid Spain; ^6^ Department of Biological Engineering Massachusetts Institute of Technology Cambridge MA 02139 USA; ^7^ Department of Biochemistry and Molecular Biology Pennsylvania State University University Park, State College PA 16802 USA; ^8^ Huck Center for Malaria Research Pennsylvania State University University Park, State College PA 16802 USA; ^9^ Wits Research Institute for Malaria Faculty of Health Sciences University of the Witwatersrand Johannesburg 2193 South Africa; ^10^ Centre for Emerging Zoonotic and Parasitic Diseases National Institute for Communicable Diseases a Division of the National Health Laboratory Service Johannesburg 2193 South Africa; ^11^ Department of Microbiology and Immunology Columbia University Irving Medical Center New York NY 10032 USA; ^12^ Center for Malaria Therapeutics and Antimicrobial Resistance Division of Infectious Diseases Department of Medicine Columbia University Irving Medical Center New York NY 10032 USA; ^13^ Swiss Tropical and Public Health Institute Kreuzstrasse 2 Allschwil 4123 Switzerland; ^14^ University of Basel Basel 4003 Switzerland; ^15^ Department of Biochemistry Stellenbosch University, Stellenbosch Matieland 7602 South Africa

**Keywords:** Antiplasmodial, Biological activity, Malaria, Medicinal chemistry, Phosphatidylinositol 4‐kinase type III beta (PI4Kβ), Repositioning

## Abstract

New compounds targeting human malaria parasites are critical for effective malaria control and elimination. Here, we pursued the imidazoquinolinone AZD0156 (MMV1580483), a human ataxia‐telangiectasia mutated (ATM) kinase inhibitor that completed Phase I clinical trials as an anticancer agent. We validated its in vitro activity against the two main forms of the *Plasmodium falciparum* parasite in the human host, viz. the asexual blood (symptomatic) stage and sexual gametocyte (transmission) stage. Resistance selection, cross‐resistance, biochemical, and conditional knockdown studies revealed that AZD0156 inhibits *P. falciparum* phosphatidylinositol 4‐kinase type III beta (*Pf*PI4Kβ), a clinically‐validated target for the treatment of malaria. Metabolic perturbations, fixed‐ratio isobolograms, killing kinetics and morphological evaluation correlated AZD0156 inhibition with other known PI4Kβ inhibitors. The compound showed favorable in vivo pharmacokinetic properties and 81% antimalarial efficacy (4 × 50 mg kg^−1^) in a *P. berghei* mouse malaria infection model. Importantly, a cleaner biochemical profile was measured against human kinases (MAP4K4, MINK1) implicated in embryofoetal developmental toxicity associated with the *Pf*PI4Kβ inhibitor MMV390048. This improved kinase selectivity profile and structural differentiation from other PI4Kβ inhibitors, together with its multistage antiplasmodial activity and favorable pharmacokinetic properties, makes AZD0156 an attractive candidate for target‐based drug repositioning against malaria via a medicinal chemistry optimization approach.

## Graphical abstract

AZD0156, an ATM kinase inhibitor in clinical development, shows promising multistage antiplasmodial activity by targeting *Plasmodium falciparum* phosphatidylinositol 4‐kinase (*Pf*PI4Kβ). With an improved specificity profile relative to other *Pf*PI4Kβ inhibitors and moderate efficacy in a *P. berghei* disease model, AZD0156 is an attractive candidate for medicinal chemistry optimization against malaria.

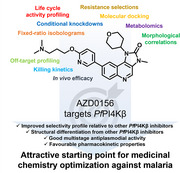

## Introduction

The efficacy of the current arsenal of clinical antimalarials, the cornerstone of parasite control, is constantly threatened by the emergence and spread of drug‐resistant strains of the most virulent human malaria parasite, *Plasmodium falciparum*.^[^ ^1^, ^2^, ^3^ ^]^ This calls for the urgent development of new chemotherapies, ideally with novel modes of action and activity against multiple stages of *P. falciparum*, to circumvent untreatable infections and contribute to malaria elimination.^[^ ^4^, ^5^ ^]^ Novel antimalarials that simultaneously target the pathogenic asexual blood stage (ABS) parasites and transmissible gametocytes will have both therapeutic and transmission‐blocking relevance, particularly if a vulnerable and essential biological pathway is targeted.^[^ ^5^, ^6^ ^]^

In this respect, due to their essentiality and expression across multiple life cycle stages, *Plasmodium* kinases are attractive as targets for the development of potential multistage‐active antimalarials.^[^ ^4^, ^7^, ^8^, ^9^ ^]^ One such example is the clinically‐validated target *Plasmodium* phosphatidylinositol 4‐kinase type III beta (PI4Kβ)^[^ ^4^, ^7^ ^]^ which catalyzes the conversion of phosphatidylinositol to phosphatidylinositol 4‐phosphate to regulate intracellular signalling and trafficking.^[^ ^10^, ^11^ ^]^ MMV390048, an aminopyridine that reached Phase II clinical trials for the treatment of malaria, was validated as a potent *Plasmodium* PI4Kβ inhibitor.^[^ ^2^ ^]^ However, embryofoetal studies prevented the further development of MMV390048 as off‐target inhibition of the mammalian PI4Kβ orthologue, in combination with the mammalian mitogen‐activated protein kinase 4 (MAP4K4) and misshapen‐like kinase 1 (MINK1), were speculated to result in developmental toxicity signals in rats. Interestingly, these toxicities were not observed in rabbits.^[^ ^12^ ^]^ Hence, despite the druggable nature of *Pf*PI4Kβ, there is a pressing need to identify new chemotypes with a cleaner selectivity and toxicity profile.

To leverage existing data and mitigate time and cost risks, the repositioning of anticancer compounds for malaria has recently attracted considerable interest.^[^ ^13^, ^14^, ^15^, ^16^ ^]^ In this context, “repositioning” refers to using an existing compound as a starting point for medicinal chemistry optimization towards treatment for a new indication, and is contrasted with “repurposing” in which a new compound is used directly to treat a different indication than the one for which it was originally developed, without chemical modification of that compound. Parallel screens of the open‐source Medicines for Malaria Venture (MMV) Pandemic Response Box (PRB) identified AZD0156 (MMV1580483) as having in vitro antiplasmodial activity across multiple stages of *P. falciparum* development, viz. the liver stages, ABS parasites and transmissible stages (gametocytes).^[^ ^17^ ^]^ This imidazoquinolinone was developed as a human ataxia‐telangiectasia mutated (ATM) kinase inhibitor and completed Phase 1 clinical trials for advanced solid tumours, alone and in combination with other anticancer treatments (https://clinicaltrials.gov/study/NCT02588105).

Here, we explored the potential of repositioning AZD0156 as a multistage antimalarial candidate through extensive mechanistic evaluation against *P. falciparum* parasites. Phenotypic approaches and biochemical profiling revealed that AZD0156 inhibits *Pf*PI4Kβ. Although this kinase has been shown to be an essential and promising multistage target,^[^ ^11^ ^]^ off‐target activities due to the conserved kinase ATP binding site have created challenges for developing selective *Pf*PI4Kβ inhibitors. Here we show that AZD0156 has minimal off‐target interactions toward other key *P. falciparum* and human kinases, unlike those previously reported for other *Pf*PI4Kβ inhibitors. This unique selectivity profile, combined with good in vitro and in vivo efficacy, positions this structurally‐differentiated chemotype as an attractive starting point for further medicinal chemistry optimization as a potential novel treatment and transmission‐blocking agent for malaria.

## Results and Discussion

### Hit Confirmation of the Multistage Activity of AZD0156

**Figure 1:**
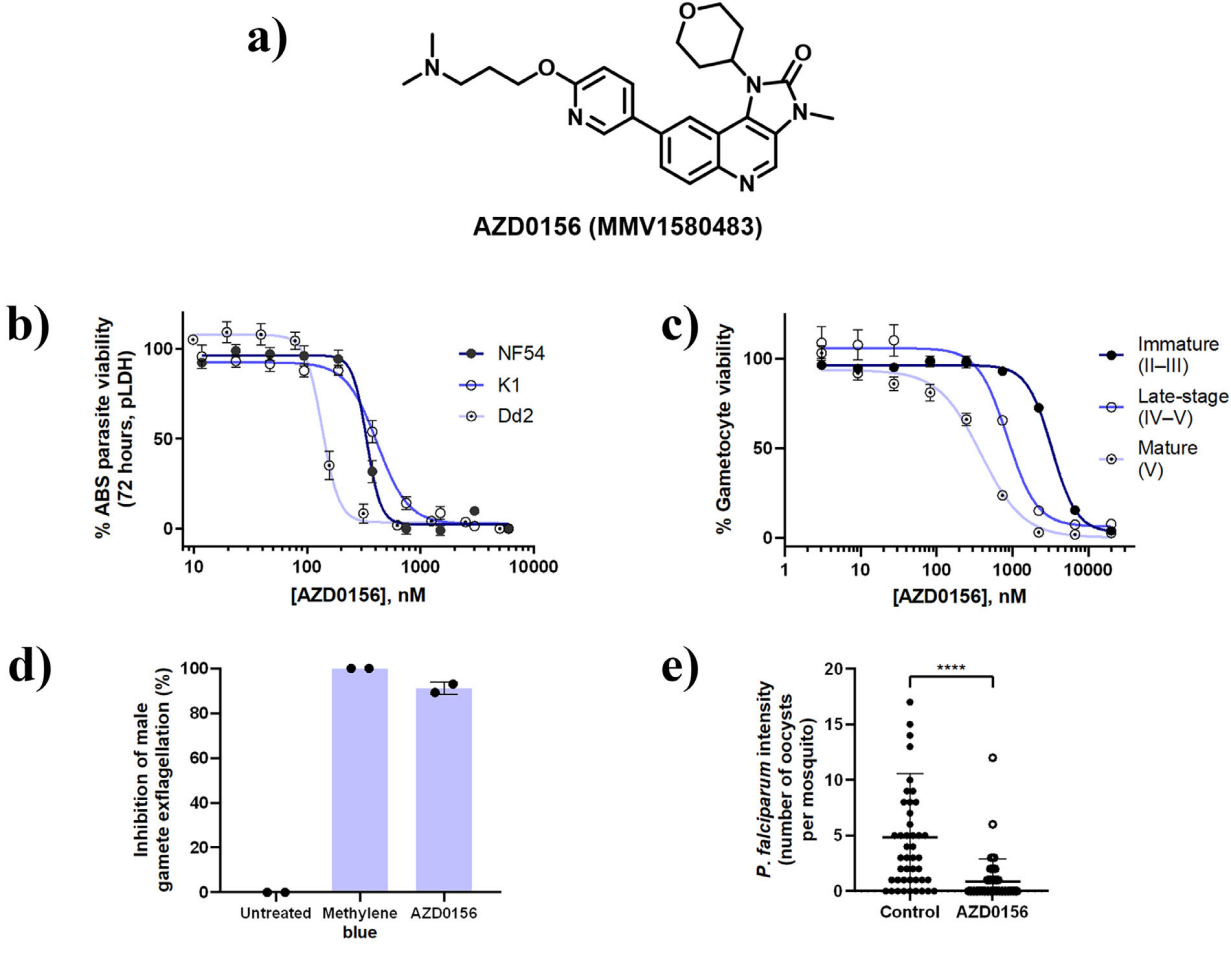
Profiling of antiplasmodial activity of AZD0156 across the *P. falciparum* life cycle. a) Structure of imidazoquinolinone AZD0156 (MMV1580483). b) ABS antiplasmodial activity of AZD0156 measured in vitro in *P. falciparum* 72 h pLDH growth inhibition assays across three strains. Dose‐response curves generated from at least three independent biological repeats with technical duplicates (mean IC_50_ value ± S.E.). Significance was determined using Mann–Whitney U tests for *Pf*NF54 versus *Pf*Dd2 (n.s.; *p* = 0.0571) and *Pf*K1 versus *Pf*Dd2 (* *p* = 0.0286). c) Effect of AZD0156 on immature (>90% stages II–III), late (>90% stages IV–V) and mature (>95% stage V) stage gametocytes using the *P. falciparum* luciferase reporter line 3D7*elo1*‐*pfs16*‐CBG99. Dose‐response curves generated from three independent biological repeats with technical triplicates (mean IC_50_ value ± S.E.). d) Male gamete viability was determined using an exflagellation inhibition assay at 2 µM. Data are from two independent biological experiments (*n *= 2, mean ± SD) relative to methylene blue at 2 µM (100%). e) Mosquito‐based standard membrane feeding assay to determine transmission‐reducing activity 8–10 days after feeding *A. coluzzii* female mosquitoes on gametocyte‐infected blood, treated with 2 µM AZD0156 for 48 h. Data are from two independent biological experiments (mean ± SD) (*n* ≥ 45, *p *< 0.0001, Mann–Whitney U test).

AZD0156 (Figure 1a) was previously identified as a singleton hit after screening the MMV pandemic response box diversity set.^[^ ^17^ ^]^ The compound was resynthesized and the reported multistage activities were confirmed (Figure 1). AZD0156 displayed submicromolar in vitro antiplasmodial activity against drug‐sensitive (*Pf*NF54 IC_50_ 335 ± 23 nM) and drug‐resistant (*Pf*K1 IC_50_ 415 ± 23 nM; *Pf*Dd2 IC_50_ 136 ± 7 nM) ABS parasites in a 72 h metabolic parasite lactate dehydrogenase (pLDH) assay (Figure 1b). Late‐stage gametocyte (>90% stages IV–V) activity on a luciferase‐reporter assay (Figure 1c; IC_50_ 943 ± 41 nM) were in alignment with previous data obtained using the PrestoBlue cell viability platform (236 ± 0 nM).^[^ ^17^ ^]^ The activity against these forms of the parasite was further interrogated on both immature (>90% stages II–III) and mature (>95% stage V) gametocytes. Interestingly, AZD0156 showed a decrease in activity against *Pf*NF54 immature gametocytes (IC_50_ 3 312 ± 76 nM) but, notably, AZD0156 showed equipotent mature gametocyte activity (IC_50_ 384 ± 37 nM) compared to ABS parasites (< two‐fold change). The decrease in activity against immature gametocytes is a phenotype rarely observed for small molecules against these gametocyte stages.^[^ ^18^ ^]^ However, the activity against mature gametocytes prompted the assessment of transmission‐blocking potential of AZD0156 against male gamete exflagellation (exflagellation inhibition assay) and oocyst formation (standard membrane feeding assay, SMFA). In line with previously‐reported transmission‐blocking activity,^[^ ^17^ ^]^ AZD0156 showed 90% inhibition of male gamete exflagellation at 2 µM (Figure 1d). Additionally, AZD0156 significantly reduced oocyst prevalence with a 56 ± 6% transmission‐blocking and 82 ± 5% transmission‐reducing activities (Figure 1e), respectively.

### *Plasmodium* PI4Kβ Is the Primary Target of AZD0156

**Figure 2:**
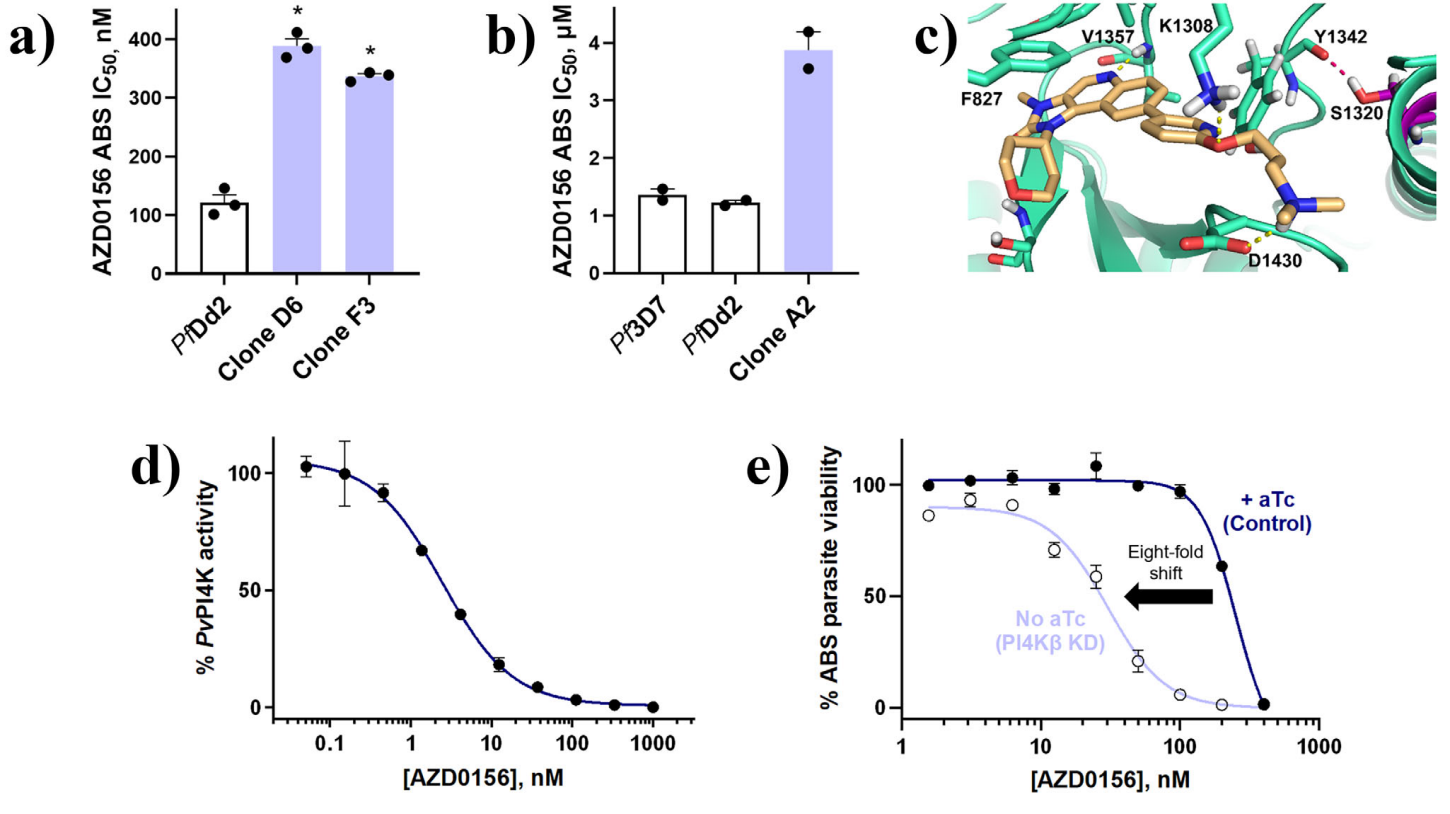
*Plasmodium* PI4Kβ is identified as the primary target of AZD0156 through resistance selection, cross‐resistance, biochemical, and conditional knockdown studies. a) Resistant clones D6 and F3 selected under AZD0156 at 3 × IC_50_ show a three‐fold loss in activity relative to the *Pf*Dd2 parental line. Mean IC_50_ values ± S.E. were calculated from three independent experiments, each with three technical repeats. Significance was determined using Mann–Whitney U tests. Comparisons are shown between *Pf*Dd2 and AZD0156‐resistant mutants; * *p* < 0.05. b) ABS clone A2, containing the A1319V mutation in *Pf*PI4Kβ selected by MMV390048, demonstrates a 3.2‐fold IC_50_ shift for AZD0156 relative to *Pf*Dd2 using the [^3^H]‐hypoxanthine incorporation assay in duplicate (mean IC_50_ value ± SD). c) Molecular docking interactions for AZD0156, typical of *Pf*PI4Kβ inhibitors in a *Pf*PI4Kβ homology model. Highlighted in purple is the position of serine 1320, which forms a hydrogen bond with tyrosine 1342 and is mutated to leucine in resistant clones D6 and F3. d) Recombinant purified *Pv*PI4Kβ is inhibited by AZD0156. Assays were carried out in the presence of 10 µM ATP and enzyme activity was measured by quantifying the amount of ADP produced during the kinase reaction using the ADP‐Glo Kinase Assay. Error bars represent ± SD for technical duplicates and a representative dose‐response curve is shown. e) Conditional knockdown (cKD) of *Pf*PI4Kβ leads to increased parasite sensitivity to AZD0156 relative to control conditions in the absence of aTc. Representative dose‐response curves are shown with error bars representing ± S.E. for technical duplicates. Results were confirmed in *n* ≥ 3 independent experiments.

Selecting for resistant mutants against ABS *P. falciparum* parasites in vitro, followed by whole‐genome sequencing, is an unbiased approach to identify resistance mechanisms and potential drug targets. Therefore, resistance selections with AZD0156 were performed by exposing 2 × 10^9^ *Pf*Dd2 parasites in two separate culture flasks to AZD0156 at 3 × IC_50_. After 22 days, a resistant population recrudesced in one flask and the bulk culture was cloned via limiting dilution. The profiled clones, D6 and F3, were ∼three‐fold less sensitive to AZD0156 (IC_50_ 389 ± 13 nM and 337 ± 5 nM, respectively) compared with the *Pf*Dd2 parental line (IC_50_ 121 ± 13 nM) (Figure 2a). Whole‐genome sequencing of clones D6 and F3 revealed a single nucleotide polymorphism in *pfpi4kβ* (PF3D7_0509800) translating to a S1320L mutation in *Pf*PI4Kβ (Figure S1A). Interestingly, the *Pf*PI4Kβ S1320L mutation was previously observed following a resistance selection with the *Hs*PI4KIIIβ and *Pf*PI4Kβ inhibitor, BQR695.^[^ ^11^ ^]^ Clone D6 additionally harboured a mutation in a putative trafficking protein (Figure S1A); however, no copy number variations (CNVs) were observed in either clone.

The clinical candidate MMV390048 also targets *Pf*PI4Kβ and resistance against MMV390048 was due to a A1319V mutation in *Pf*PI4Kβ that caused a four‐fold IC_50_ shift for MMV390048 relative to the *Pf*Dd2 parental line.^[^ ^19^ ^]^ Notably, the position of the A1319V mutation in *Pf*PI4Kβ is adjacent to the S1320L mutation selected by AZD0156. This *Pf*PI4Kβ A1319V mutant parasite line (clone A2) was subsequently employed to cross‐screen for the potential of AZD0156 resistance, whereupon a differential susceptibility relative to *Pf*Dd2 was also observed with an average fold IC_50_ shift for AZD0156 of 3.2 (Figure 2b), tracking with the phenotypic shifts of the *Pf*PI4Kβ‐S1320L mutants.

Based on a *Pf*PI4Kβ homology model,^[^ ^20^ ^]^ S1320L is located in the ATP‐binding site between the hinge binding region and affinity pocket (Figure 2c). Interestingly, the S1320L mutation results in loss of a hydrogen bond that is typically formed in the wild type protein between S1320 and Y1342, and this could distort the binding site in the mutated protein relative to the wild type, giving rise to the resistant phenotype. Molecular docking predictions in the wild type isoform show that AZD0156 forms key interactions with the phosphate‐binding loop (P‐loop, F827), catalytic site (K1308), hinge‐binding region (V1357), ribose pocket (S1362), and affinity pocket (D1430) (Figures 2c and S1B).^[^ ^20^ ^]^

To confirm *Pf*PI4Kβ as the target of AZD0156 in vitro, we used a *P. vivax* PI4Kβ recombinant enzyme assay, based on a 97% sequence homology between the *Pf*PI4K and *Pv*PI4K ATP‐binding site and catalytic region and known difficulties for expressing *Pf*PI4Kβ.^[^ ^15^ ^]^ Against *Pv*PI4Kβ, we recorded an IC_50_ value of 5 ± 2 nM for AZD0156 (Figure 2d), directly comparable to previously‐validated *Pf*PI4Kβ inhibitors MMV390048, UCT943 and sapanisertib, all of which recorded IC_50_ values <5 nM in this assay.^[^ ^4^, ^19^, ^21^ ^]^

We also probed the effect of PI4Kβ knockdown on parasite sensitivity to AZD0156 using an engineered *Pf*PI4Kβ conditional knockdown (cKD) parasite line.^[^ ^15^ ^]^ In this assay, *Pf*PI4Kβ translation is reduced by the removal of anhydrotetracycline (aTc) through the TetR (Tet repressor protein)/DOZI (development of zygote inhibited)‐RNA aptamer module. Thus, a low aTc concentration constitutes a conditional *Pf*PI4Kβ knockdown.^[^ ^22^, ^23^ ^]^ The cKD of *Pf*PI4Kβ led to eight‐fold increased parasite sensitivity to AZD0156 (Figure 2e, IC_50_ 30 ± 2 nM and 252 ± 40 nM, respectively), comparable to a > 10‐fold shift previously reported for the *Pf*PI4Kβ inhibitor, sapanisertib.^[^ ^15^ ^]^ This is consistent with decreased target protein requiring less compound for parasite growth to be inhibited. Thus, in line with observations for other validated *Pf*PI4Kβ inhibitors, these data substantiated *Pf*PI4Kβ as the primary target of AZD0156.

### AZD0156 has a Cleaner Selectivity Profile for *Pf*PI4Kβ Compared to Other PI4Kβ Inhibitors

Off‐target activity is a significant challenge when developing kinase inhibitors due to the conserved ATP‐binding site of this superfamily of enzymes. ATM kinases propagate an extensive signalling cascade in response to DNA damage; hence, ATM kinase inhibitors are frequently used with radiation therapy or other DNA‐damaging agents.^[^ ^24^, ^25^, ^26^ ^]^ *Plasmodium* parasites have a divergent cell cycle progression profile evident from a lack of canonical cell cycle checkpoint proteins, such as Rb and p53 proteins, and ATM and ATR kinases.^[^ ^27^, ^28^ ^]^ *Pf*PI3K has also been identified as a potential kinase inhibitor target of AZD0156^[^ ^15^ ^]^ and inhibition data for AZD0156 against the human homologue of *Pf*PI4K, *Hs*PI3K, have already been published (0.32 µM, 1.8 µM, 1.1 µM, and 0.27 µM for the α, β, γ, and δ isoforms of *Hs*PI3K, respectively^[^ ^26^ ^]^); however, this did not preclude the compound from entering and completing Phase I clinical development.

**Figure 3:**
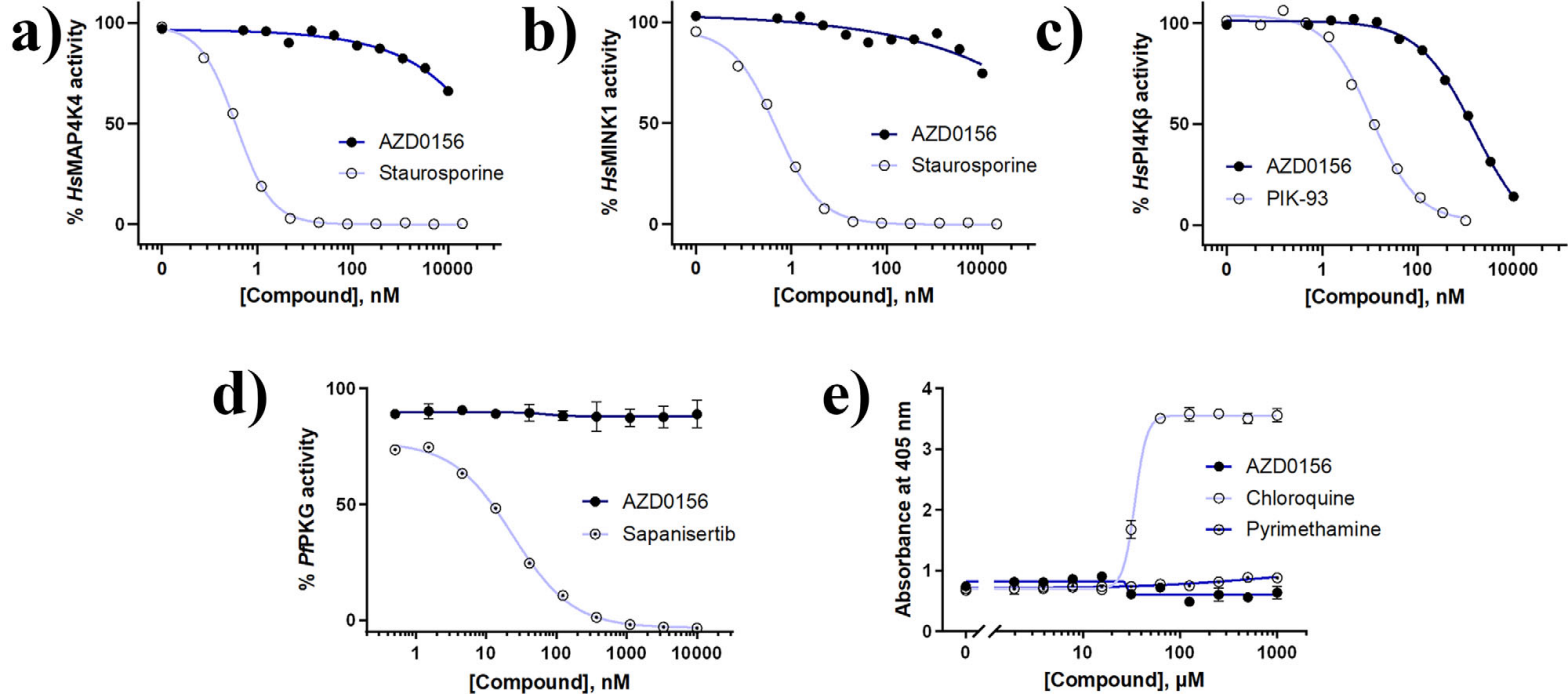
AZD0156 displays improved specificity for *Pf*PI4Kβ relative to other PI4Kβ inhibitors. Biochemical inhibition of AZD0156 against putative off‐targets (a) *Hs*MAP4K4 and (b) *Hs*MINK1, both demonstrating <50% inhibition at 10 µM relative to the positive control (staurosporine) for those assays, and (c) *Hs*PI4Kβ relative to its control (PIK‐93). d) In vitro recombinant enzyme activity of AZD0156 against *Pf*PKG. A representative dose‐response curve is shown. Assays were carried out in the presence of 10 µM ATP and enzyme activity was measured by quantifying the amount of ADP produced during the kinase reaction using the ADP‐Glo Kinase Assay. Error bars represent ± SD for technical duplicates. e) The inhibition of synthetic haemozoin (β‐haematin) formation using a detergent‐mediated assay, indicated by the increased absorbance at 405 nm, for AZD0156 relative to the positive control chloroquine and the negative control pyrimethamine. Dose‐response curve generated from three technical duplicates (mean IC_50_ value ± S.E.).

In light of a report speculating that *Pf*PI4Kβ inhibitors, specifically MMV390048, may interact deleteriously with mammalian MAP4K4 and MINK1, in combination with *Hs*PI4Kβ, leading to embryofoetal toxicity (teratogenicity),^[^ ^12^ ^]^ we evaluated biochemical activity against these human kinases. For *Hs*MAP4K4 and *Hs*MINK1, AZD0156 showed <50% enzyme inhibition at 10 µM, compared to nanomolar inhibition by the reference compound, staurosporine, a promiscuous ATP‐competitive kinase inhibitor (Figure 3a,b). This was a promising improvement on the inhibition data previously measured for MMV390048, viz. 0.8 µM and 0.7 µM against *Hs*MAP4K4 and *Hs*MINK1, respectively.^[^ ^12^ ^]^ Although AZD0156 showed partial inhibition of the *Pf*PI4Kβ orthologue, *Hs*PI4Kβ (IC_50_ 1.22 µM, Figure 3c), which is comparable to that previously reported for MMV390048 (1 µM),^[^ ^12^ ^]^ these are encouraging data which indicate that the imidazoquinolinone scaffold has a biochemical profile that is distinct from other *Pf*PI4K inhibitors. This improved selectivity profile provides an attractive starting point for medicinal chemistry optimization of this chemotype.

Recent work has demonstrated that *Pf*PI4Kβ inhibitors may also target other enzymes or pathways in *P. falciparum* in a dual inhibitor fashion; for example, the anticancer kinase inhibitor sapanisertib targets both PI4Kβ and *P. falciparum* cGMP‐dependent protein kinase (*Pf*PKG).^[^ ^15^ ^]^ Although such polypharmacology is an attractive attribute in some respects, finding the balance between potency and on‐target activity can be challenging in target‐guided drug discovery programmes for dual inhibitors. AZD0156 was found not to inhibit *Pf*PKG (IC_50_ > 10 µM, Figure 3d) in contrast to the known *Pf*PI4Kβ/*Pf*PKG inhibitor, sapanisertib. A subset of naphthyridine‐based *Pf*PI4Kβ inhibitors may also target the haem detoxification pathway in ABS parasites.^[^ ^29^ ^]^ However, unlike that series, AZD0156 was found not to inhibit β‐haematin (synthetic haemozoin) formation using an extracellular detergent‐mediated indicator assay, relative to the standard β‐haematin and haemozoin formation inhibitor chloroquine, but rather tracked with the negative control, pyrimethamine (Figure 3e).

Although AZD0165 may not necessarily have direct off‐target effects on the human and *Plasmodium* orthologues as presented here, future studies would have to include off‐target evaluations against other kinases, such as *Hs*ATM. Fortunately, the relatively lower dose coupled with a shorter length of treatment required to deal with an acute malaria infection, compared to those required for cancer, could potentially alleviate some off‐target concerns.

### AZD0156 Treatment Associates With PI4Kβ‐Targeting Phenotypes and Killing Kinetics

**Figure 4:**
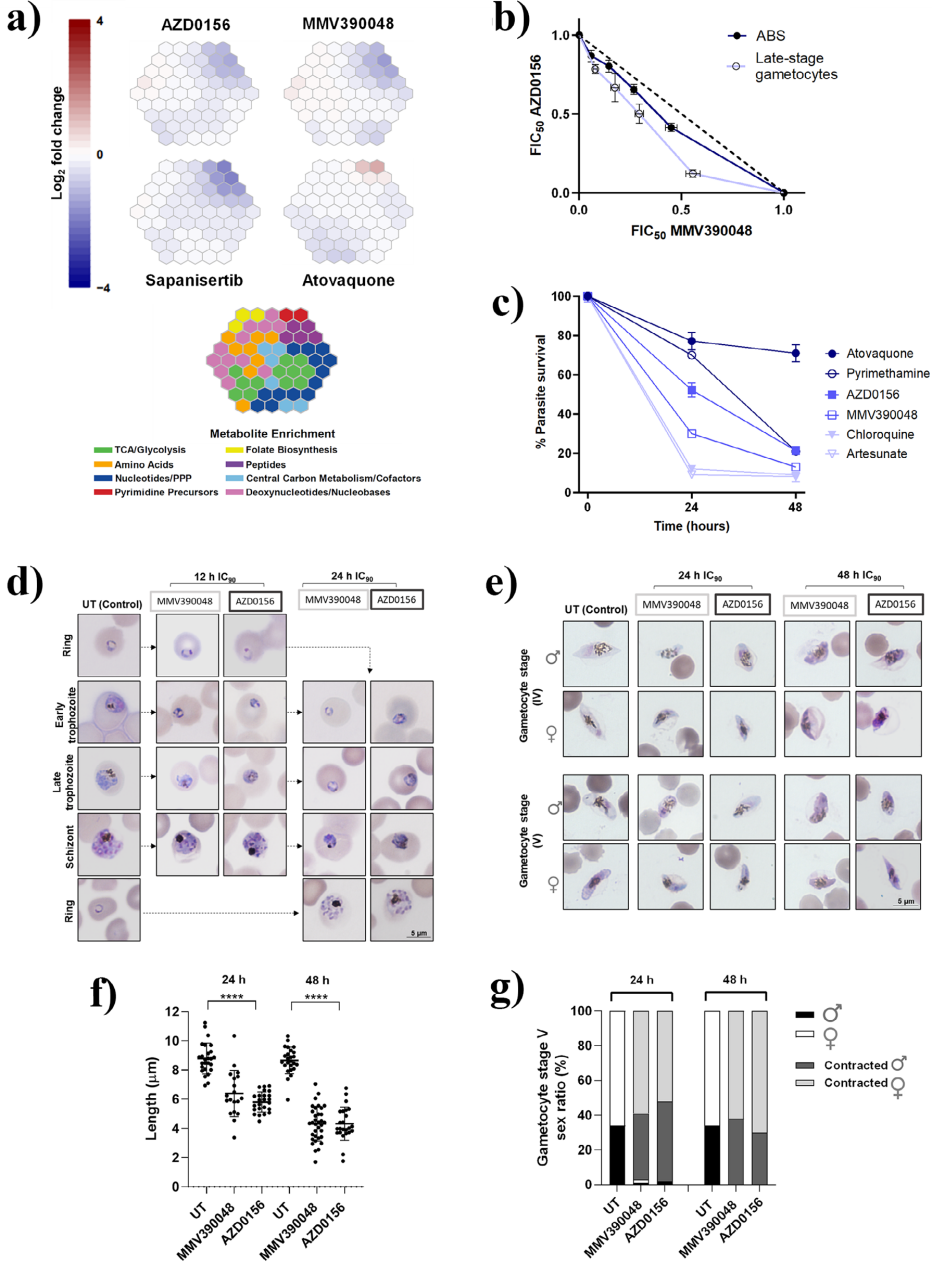
AZD0156 displays phenotypic responses and killing kinetics similar to other *Pf*PI4Kβ inhibitors. a) Metabolomic fingerprint analysis after treatment of *P. falciparum* trophozoites for 2.5 h with *Pf*PI4Kβ inhibitors at 10 × IC_50_. Data are presented using 2D hexagonal maps (‘metaprints’) wherein 113 targeted metabolites are represented as metabolite clusters, separated into eight known metabolic pathways. b) Fixed‐ratio isobologram analysis for AZD0156 with MMV390048 against ABS and late‐stage gametocytes (>90% IV–V). Data represent the mean fractional inhibitory concentration (FIC_50_) for four drug combination ratios of three biological replicates each performed in technical duplicates (ABS) or triplicates (late‐stage gametocytes) with error bars representing ± S.E. c) AZD0156 has moderate killing kinetics relative to known antimalarial drugs as demonstrated in a 48 h PRR assay, tracking with *Pf*PI4Kβ inhibitor MV390048. Giemsa‐stained thin smear morphological and stage‐specific evaluation of *P. falciparum* (d) ABS (ring, early/late trophozoite, and schizont) parasites for 12 or 24 h and (e) stage IV and stage V gametocytes for 24 h or 48 h under hypoxic conditions following MMV390048 and AZD0156 IC_90_ treatment. Giemsa‐stained thin smear morphological and stage‐specific evaluation of *P. falciparum* stage IV and stage V gametocytes with MMV390048 and AZD0156 at IC_90_‐treated stage IV and V gametocytes for 24 h and 48 h under hypoxic conditions at 37°C: f) mean gametocyte length (µm) quantified for 24 and 48 h treated and untreated (UT) populations (*n* ≥ 20, *p *< 0.0001, one sample *t*‐test), g) and percentage male‐to‐female distribution for the same population.

An established metabolomics approach^[^ ^30^ ^]^ was used to assess the impact of AZD0156 on parasite biochemical or metabolic pathways relative to other known *Plasmodium* kinase inhibitors. Drug‐sensitive *Pf*3D7 trophozoites were treated for 2.5 h at 10 × IC_50_ with AZD0156, MMV390048 and sapanisertib, and the control compound atovaquone (a cytochrome *bc*_1_ complex inhibitor). The metabolic response to each compound following treatment exposure was determined by liquid chromatography‐coupled mass spectrometry. Metabolite changes compared to the untreated control revealed drug‐induced disruptions to key metabolic pathways. The metabolic fingerprint of AZD0156 was distinct from the control, atovaquone. The AZD0156 metaprint is more similar to that of known *Pf*PI4Kβ inhibitor MMV390048 but less so to sapanisertib as a *Pf*PI4Kβ/*Pf*PKG dual inhibitor, again confirming the observed differentiation of AZD0156 from these other inhibitors and supporting its more selective *Pf*PI4Kβ inhibition (Figure 4a).^[^ ^15^ ^]^ Illustrated another way, principal component analysis and a heatmap correlation matrix (Figure S2), based on metabolic shifts, show the clustering of AZD0156 with other known *Pf*PI4Kβ inhibitors rather than with compounds known to inhibit other targets. This metabolic phenotype associated with *Pf*PI4Kβ inhibitors results in a decrease in haemoglobin‐derived peptides and, while not inhibiting haemozoin formation as implied in Figure 3e above, suggests some disruption of haemoglobin catabolism via other pathways, although it should be noted that this effect is often observed for antiplasmodial agents of other classes if the test compound concentration is high enough.^[^ ^30^ ^]^

AZD0156 also behaved similarly to MMV390048 in fixed‐ratio isobologram analysis on the two main life cycle stages of *P. falciparum*. AZD0156 combined with MMV390048 showed additive/indifferent interactions for both ABS parasites (ΣFIC_50_ 0.9) and late‐stage gametocytes (ΣFIC_50_ 0.8) (Figure 4b). Additionally, the two compounds reacted similarly when combined with dihydroartemisinin (DHA) which acts through a nonkinase‐related mechanism. When AZD0156 treatment was combined with DHA (Figure S3A), or MMV390048 together with DHA (Figure S3B), the combination was, in each instance, antagonistic for ABS parasites (ΣFIC_50_ 1.5 for both combinations) and synergistic on late‐stage gametocytes (ΣFIC_50_ 0.9 for AZD0156 with DHA and ΣFIC_50_ 0.5 for MMV390048 with DHA).^[^ ^31^ ^]^

AZD0156 showed moderate killing kinetics in the *Pf*3D7 strain over 48 h in an in vitro parasite reduction ratio (PRR) assay relative to known antimalarial drugs (Figure 4c). Quantitatively, while AZD0156 exhibited a faster rate of kill compared to atovaquone and pyrimethamine, the in vitro logPRR for AZD0156 indicated a slower rate of kill of 1.3 compared to other known *Pf*PI4Kβ inhibitors, MMV390048 (logPRR 2.7)^[^ ^19^, ^21^ ^]^ and UCT943 (logPRR 2.5), again suggesting some differentiation from existing *Pf*PI4Kβ inhibitors.^[^ ^21^ ^]^ However, because the rate of kill depends on both the mode of action and the physicochemical properties of the compound, the killing kinetics of this chemotype could potentially change during medicinal chemistry optimization. As antimalarial agents are typically used in combination, compounds with a variety of killing rates are useful in deciding the optimal combination regimen.

Both MMV390048 and UCT943 have been noted to present lag phases targeting late trophozoite stages and schizogony.^[^ ^19^ ^]^ For AZD0156, this was confirmed to be the case through morphological evaluation, which showed minimal effect on ring‐stage parasites with a 12 h treatment. However, a 24 h treatment completely halted trophozoite development, with treated trophozoites unable to enter schizogony (Figures 4d and S4). This rate of action of AZD0156 aligns with transcriptomic evaluations showing that *Pf*PI4Kβ expression peaks during early trophozoite formation and schizogony,^[^ ^32^ ^]^ suggesting a stage‐specific phenotype that correlates to the moderately slow rate of action previously observed for MMV390048.^[^ ^19^ ^]^

Further morphological stage‐ and sex‐specific evaluation of gametocytes treated with AZD0156 at IC_90_ for 24 and 48 h showed that gametocytes were evidently compromised (Figure 4e). Both stage IV and V gametocytes were significantly contracted (mean untreated length of 8.8 ± 0.2 µm versus AZD0156‐treated length of 5.4 ± 0.4 µm, *n *≥ 20, *p *< 0.0001, and one sample *t*‐test, Figure 4f) already after 24 h treatment. Treated stage IV gametocytes could not mature to stage V over a 48 h treatment (Figure 4e). AZD0156 treatment affected both male and female gametocytes similarly, with no change in the sex ratio (Figure 4g). The “contracted” phenotype observed due to AZD0156 treatment correlates with that seen after MMV390048 treatment. These gametocytes are not viable and significantly metabolically compromised (decreased mitochondrial respiration as indicated by MitoTracker Red CMXRos, *n* = 89, *p* = 0.012, Mann–Whitney U test, Figure S5). The correlation of the effect of AZD0156 and MMV390048 phenotypic evaluation of gametocytes corroborates *Pf*PI4Kβ as the target of AZD0156 in both ABS and gametocytes. The specificity of AZD0156 toward trophozoites and late‐stage (IV–V) gametocytes coincides with the core roles of *Pf*PI4Kβ within various developmental stages including DNA replication and cytokinesis^[^ ^33^ ^]^ required in the ABS, as well as chromatin condensation for egress during gamete formation.^[^ ^9^, ^34^ ^]^ These findings are consistent with previous studies showing that *Pf*PI4Kβ is a highly‐druggable multistage target in *P. falciparum* parasites.^[^ ^9^, ^19^, ^35^ ^]^

### AZD0156 Shows Favorable Cytotoxicity and Pharmacokinetic Properties, and Moderate in Vivo Antimalarial Efficacy

As an indicator of potential cytotoxicity, AZD0156 was screened against the human HepG2 hepatic cell line at 2 µM for which 96% cell viability was obtained, suggesting a favorable cytotoxicity profile. A more detailed cytotoxicity study was conducted against the Chinese hamster ovary (CHO) cell line, for which AZD0156 treatment yielded an IC_50_ value of 11.7 µM. This results in favorable selectivity indices of >25‐fold relative to the whole‐cell ABS potencies measured against the three different strains as presented in Figure 1b. A lack of cardiotoxicity, through minimal interaction with the hERG potassium channel at low micromolar concentrations, has also previously been reported for AZD0156 (IC_50_ > 30 µM).^[^ ^17^ ^]^ This finding further supports a favorable toxicity profile for this compound in the context of potential repositioning for malaria. Furthermore, AZD0156 was highly soluble at 195 µM in PBS (pH 6.5), compared to some other PI4Kβ inhibitors for which notably low aqueous solubility has been recorded.^[^ ^21^, ^36^ ^]^

When administered intravenously in mice, the blood clearance of AZD0156 was low (Table S1). This is consistent with the high in vitro metabolic stability observed for hepatic metabolism of this compound, viz. CL_int, app_ (mL min^−1^ kg^−1^) < 11.6, < 11.6 and < 11.6 for human, rat, and mouse‐derived liver microsomes, respectively. The plasma volume of distribution at steady state (*V*_ss_) was moderate, suggesting that the compound distributes and accumulates in organ tissues. This is expected for a basic compound due to partitioning into cell membranes associated with acidic phospholipids.^[^ ^21^ ^]^ Consequently, the half‐life of AZD0156 was moderate at approximately 4 h; this will require further optimization to achieve the desired half‐life of >4 h for a single‐dose cure.^[^ ^37^ ^]^ Though not previously reported in mice, this value correlated with half‐life measurements in rats.^[^ ^26^ ^]^ Oral bioavailability was good, at almost 50%, which is encouraging in terms of potentially achieving a single‐dose treatment and cure for malaria that aims to boost patient compliance in resource‐limited regions of the world.

Finally, when AZD0156 was dosed orally at 4 × 50 mg kg^−1^ in the *P. berghei* malaria infection model, an 81% reduction in parasitaemia was achieved (for comparison, 99.9% and 99% reduction in parasitaemia were achieved for clinical control compounds chloroquine and artesunate, respectively, in the same model at 4 × 30 mg/kg^[^ ^38^ ^]^), indicating an important pharmacology proof‐of‐concept to motivate optimization of this compound as an oral drug for humans as a component of combination therapy for the treatment of malaria.

## Conclusion

Developing new antimalarial compounds that target multiple stages of the human malaria parasite is critical to managing and reducing malaria cases and deaths effectively. Our findings demonstrate that the imidazoquinolinone AZD0156 (MMV1580483), a human ATM kinase inhibitor that completed Phase I clinical trials as a treatment for cancer, exhibits significant in vitro ABS and transmission‐blocking activity against *P. falciparum*, the most virulent human malaria parasite. Resistance selection, cross‐resistance, biochemical, and conditional knockdown studies identified *Pf*PI4Kβ, a well‐established and clinically‐validated drug target for malaria, as the molecular target of AZD0156. These data were supported by metabolomic perturbation analysis, fixed‐ratio isobolograms, and in vitro PRR assays, all of which phenotypically highlighted the similarity of AZD0156 to other known *Pf*PI4Kβ inhibitors. Further profiling revealed a lack of effective biochemical inhibition of *Hs*MAP4K4 and *Hs*MINK1, both of which have been speculated to be associated with the toxicological signals associated with MMV390048, another *Pf*PI4Kβ inhibitor. In that regard, AZD0156 does not display a polypharmacological profile associated with targeting other *P. falciparum* kinases or metabolic pathways previously observed with other *Pf*PI4Kβ inhibitors. The advantage of this specificity for a single kinase target may contribute to the minimal off‐target activity measured against mammalian kinases. These cleaner toxicity profiles suggest that this chemotype may hold fewer developmental risks. However, activity against the human anticancer target for which the compound was initially developed (ATM kinase), and risk for resistance development, will have to be closely monitored. In addition to its favorable cytotoxicity, physicochemical and pharmacokinetic properties, AZD0156 showed moderate in vivo efficacy in a *P. berghei* mouse malaria infection model. Taken together, these data demonstrate that AZD0156 is an attractive starting point for repositioning as an antiplasmodial lipid kinase inhibitor via structure‐guided medicinal chemistry optimization, with the potential to yield molecules for much‐needed therapeutic intervention against the scourge of malaria.

## Supporting Information

The authors have provided additional information in the Supporting Information, viz. supplementary figures, experimental section, and computational methods.

## Conflict of Interests

At the time of conducting this project and writing the manuscript, authors V.F. and J.G. were GSK employees and report ownership of GSK shares. No specified compensation was given to the authors in response to the development of this article. The remaining authors declare no conflict of interest.

## Supporting information



Supplementary Information

## Data Availability

The data that support the findings of this study are available from the corresponding authors upon reasonable request.
