# Supplementary material for: The ATM Kinase Inhibitor AZD0156 Is a Potent Inhibitor of Plasmodium Phosphatidylinositol 4‐Kinase (PI4Kβ) and Is an Attractive Candidate for Medicinal Chemistry Optimization Against Malaria
Source: Angew Chem Int Ed Engl. 2025 May 15;64(28):e202425206. doi: 10.1002/anie.202425206 (PMC12232896; doi:10.1002/anie.202425206)
Supplement: Supplementary file 1 — Supplementary Information [file ANIE-64-e202425206-s001.docx]

**The ATM Kinase Inhibitor AZD0156 Is a Potent Inhibitor of *Plasmodium* Phosphatidylinositol 4-Kinase (PI4Kβ) and Is an Attractive Candidate for Medicinal Chemistry Optimization Against Malaria**

John G. Woodland^1,2,3+^, Dina Coertzen^4,5+^, Kathryn J. Wicht^1,2,3^, Virginia Franco Hidalgo^6^, Charisse Flerida A. Pasaje^7^, Luiz C. Godoy^7^, Tarrick Qahash^8,9^, Mmakwena M. Mmonwa^1^, Godwin A. Dziwornu^1^, Lynn Wambua^2,3^, Sarah Harries^3^, Constance M. Korkor^3^, Mathew Njoroge^1^, Liezl Krugmann^1^, Dale Taylor^1^, Meta Leshabane^4,5^, Henrico Langeveld^4,5^, Tayla Rabie^4,5^, Janette Reader^4,5^, Mariëtte van der Watt^5^, Nelius Venter^10,11^, Erica Erlank^10,11^, Ayesha S. Aswat^10,11^, Lizette L. Koekemoer^10,11^, Tomas Yeo^12,13^, Jin H. Jeon^12,13^, David A. Fidock^12,13^, Francisco Javier Gamo^6^, Sergio Wittlin^14,15^, Jacquin C. Niles^7^, Manuel Llinas^8,9^, Lauren B. Coulson^1,2^, Lyn-Marié Birkholtz,^4,5,16*^ and Kelly Chibale^1,2,3*^

| **SUPPORTING INFORMATION**  **1. Supplementary INFORMATION AND figures**  **2. Experimental section**  **3. Computational methods** |
| --- |

**1. SUPPLEMENTARY INFORMATION AND FIGURES**

**
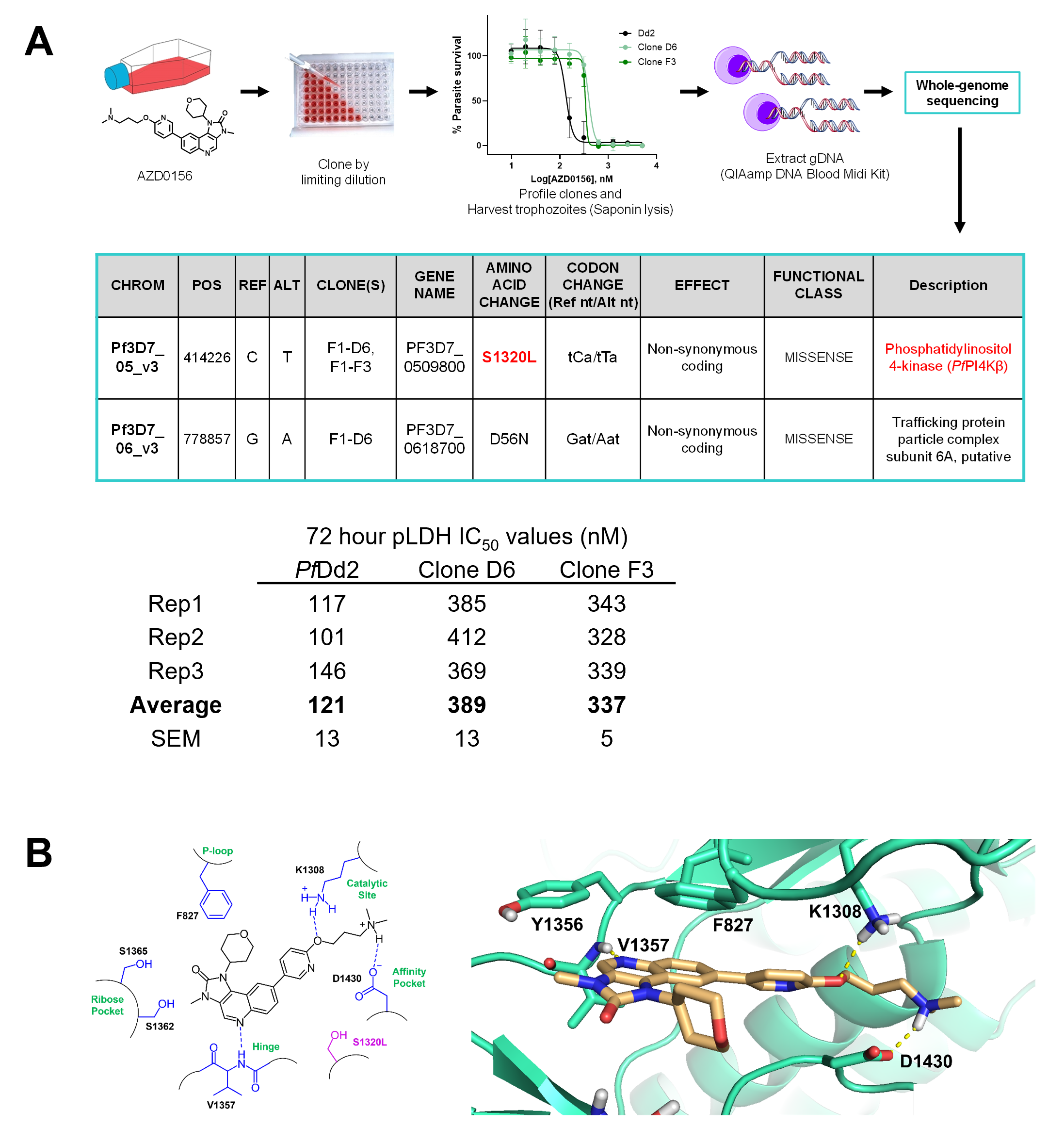
**

**Figure S1.** (**A**) Resistance selection workflow and whole-genome sequencing of clones D6 and F3 that were selected with AZD0156. Mutation S1320L in *Pf*PI4Kβ was observed in both clones, supporting this kinase as the primary target of AZD0156. No CNVs were found in either clone. Clones D6 and F3 show a ~3-fold shift in IC_50_ value relative to the parental line, *Pf*Dd2. Samples D6 and F3 had mean whole-genome coverages of 35× and 50×, respectively. (**B**) AZD0156 modelled in the ATP-binding site of the *Pf*PI4Kβ homology model showing the typical interactions of an ATP-competitive inhibitor.

**
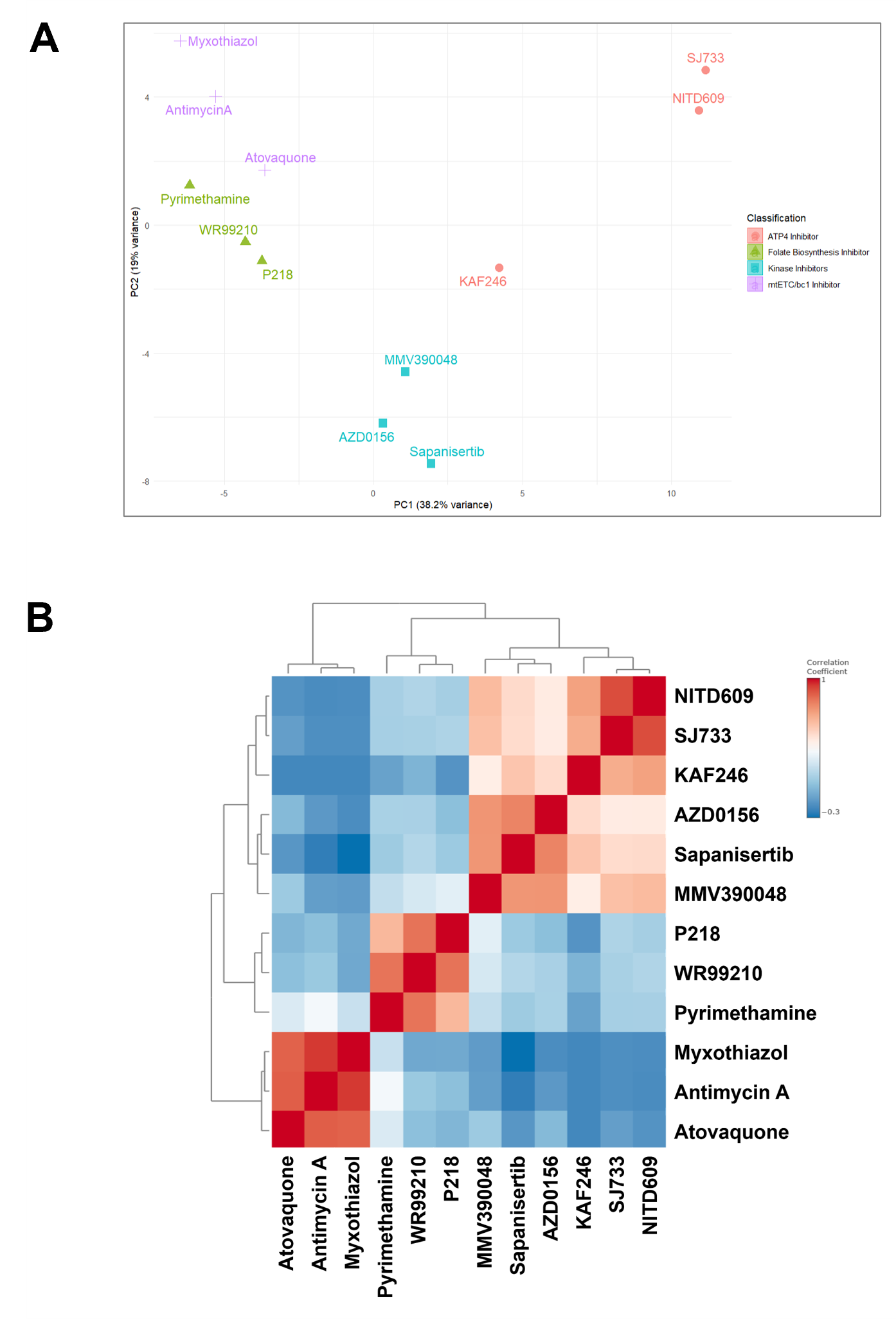
**

**Figure S2.** (**A**) Principal component analysis (PCA) of twelve drugs across four different targets and their metabolic shifts. AZD0156 clusters closest to other known *Pf*PI4Kβ inhibitors such as sapanisertib and MMV390048. (**B**) Correlation heatmap of the same twelve drugs and four targets, with clustering analysis again showing that AZD0156 groups closely with other known *Pf*PI4Kβ kinase inhibitors such as sapanisertib and MMV390048.

**
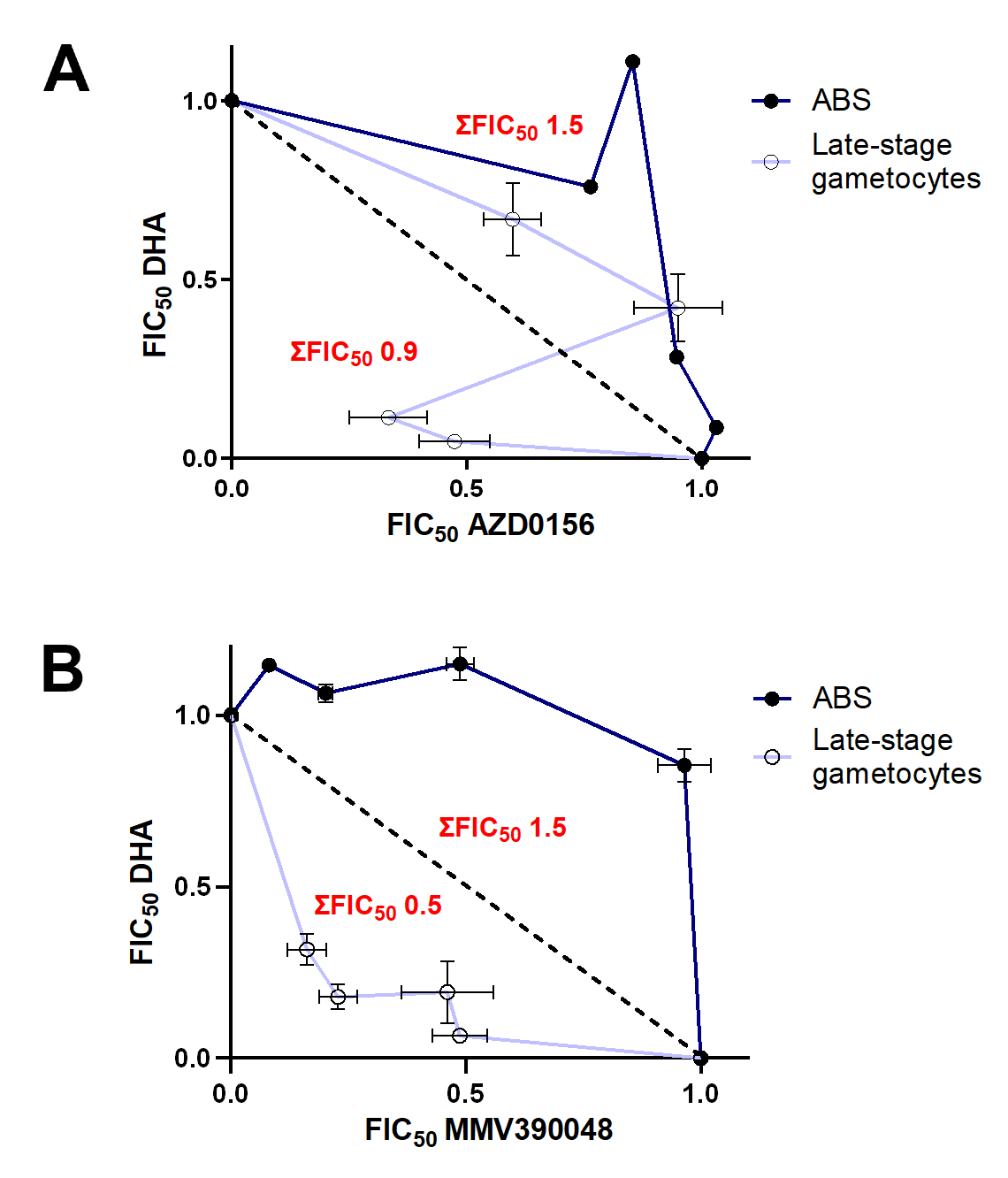
**

**Figure S3.** (**A**) Fixed-ratio isobologram analysis of AZD0156 and dihydroartemisinin (DHA) against *P. falciparum* ABS parasites and late-stage gametocytes (> 90% IV–V). Data represent a single biological replicate (*n* = 1) for ABS parasites and three biological replicates (*n* = 3) for late-stage gametocytes, performed in technical duplicates of mean fractional inhibitory concentration (FIC_50_) values ± S.E. (**B**) Fixed-ratio isobologram analysis of MMV390048, a *Pf*PI4Kβ inhibitor, and DHA against *P. falciparum* ABS parasites and late-stage gametocytes (> 90% IV–V). Data represent three biological replicates, performed in technical duplicates of mean fractional inhibitory concentration (FIC_50_) values ± S.E.


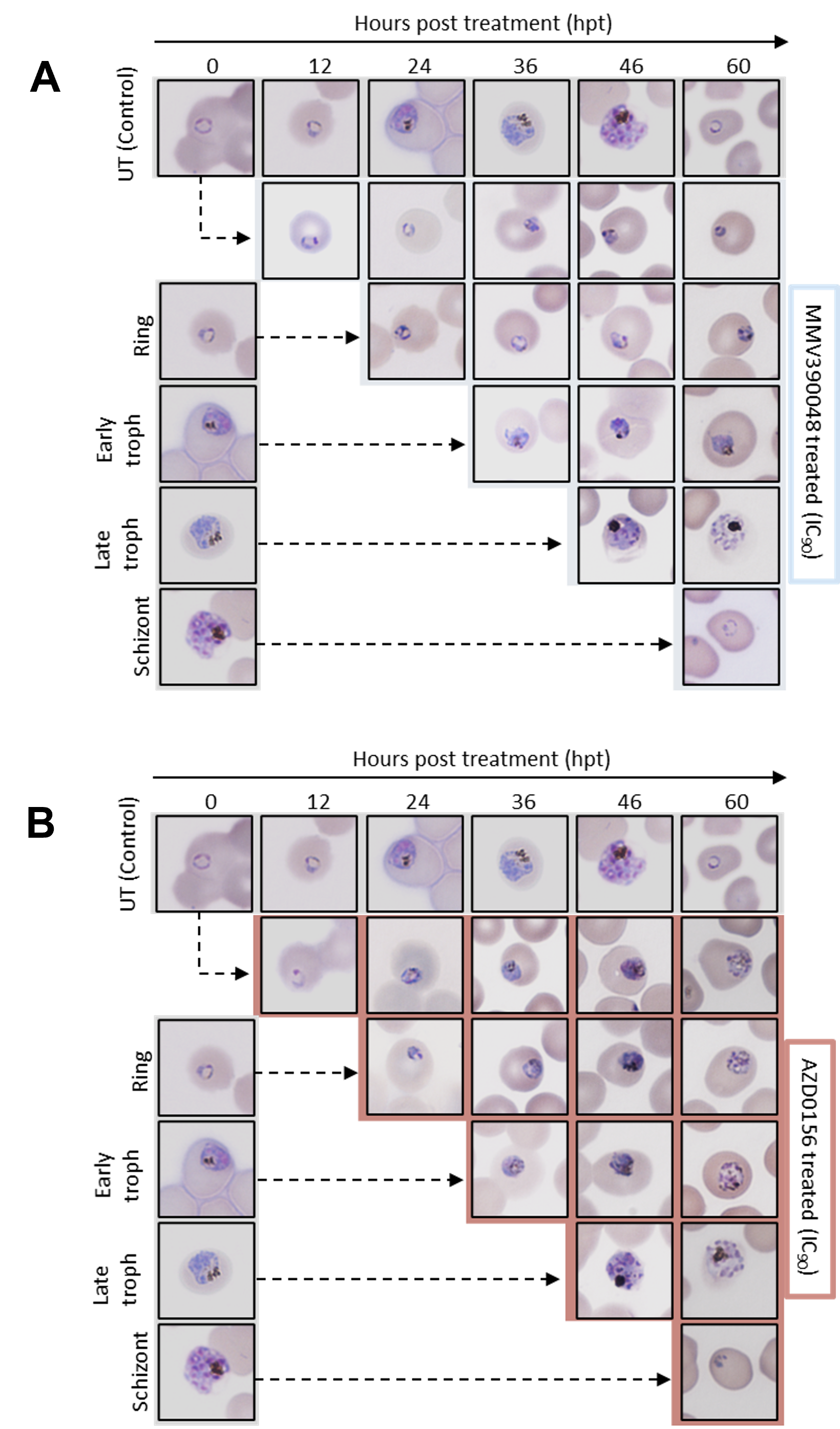


**Figure S4**. Morphological evaluation of *P. falciparum* ABS parasites following treatment with (**A**) MMV390048 and (**B**) AZD0156 IC_90_ (*n* = 1). Parasites were treated under hypoxic conditions at 37°C at 0, 12, 24, 36, 46 and 60 h post invasion. Sampling was performed every 12 h, and parasite stage distribution and morphology were evaluated using Giemsa-stained thin smears.

**
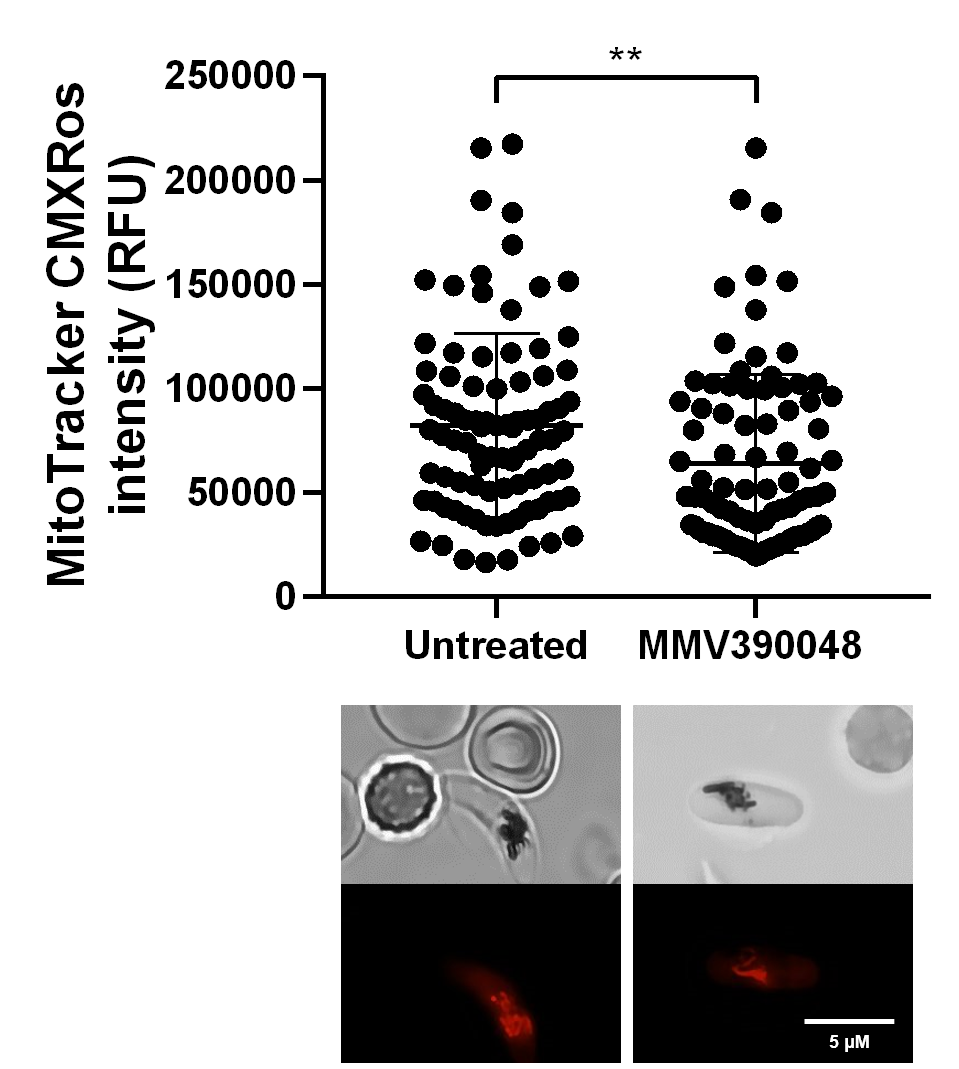
**

**Figure S5.** Viability evaluation of MMV390048 treated late-stage gametocytes (> 90% IV–V) at IC_90_ for 24 h under hypoxic conditions at 37°C using MitoTracker Red CMXRos (*n* = 89, *p* = 0.0012, Mann-Whitney U test).

**Table S1.** *In vivo* mouse pharmacokinetics for AZD0156.

| **Species and route of administration** | **Balb/C mouse  (48 h), p.o.** | **Balb/C mouse  (48 h), intravenous** |
| --- | --- | --- |
| **Formulation** | 0.5% HPMC in water  with 0.2% Tween80 | DMA/PEG/PPG  10:30:60 |
| **Dose** | 10 mg/kg | 3 mg/kg |
| ***T*_1/2_** | 3.5 h | 4.1 h |
| ***C*_max_** | 3 μM |  |
| ***V*_d_, *V*_ss_** |  | 4.8 L/kg, 3.5 L/kg |
| **AUC** | 853 min.μM | 512 min.μM |
| **Clearance** |  | 15.3 mL/min/kg |
| **Bioavailability** | 49.8% |  |

**2. EXPERIMENTAL SECTION**

**2.1. Chemistry**

AZD0156 was synthesised according to methods previously described.^[1]^ Characterisation: ^1^H NMR (300 MHz, DMSO-*d_6_*) δ 8.87 (s, 1H), 8.64 (s, 1H), 8.41 (s, 1H), 8.24–8.08 (m, 2H), 7.93 (d, *J* = 8.9 Hz, 1H), 6.97 (d, *J* = 8.6 Hz, 1H), 5.30–4.96 (m, 1H), 4.36 (t, *J* = 6.6 Hz, 2H), 4.10–4.04 (m, 2H), 3.66–3.53 (m, 2H), 3.51 (s, 3H), 2.78–2.67 (m, 2H), 2.37 (t, *J* = 7.1 Hz, 2H), 2.16 (s, 6H), 1.98–1.81 (m, 4H). ^13^C NMR (151 MHz, DMSO­­-*d_6_*) δ 163.64, 153.34, 145.72, 144.35, 138.35, 135.17, 133.75, 131.87, 129.26, 128.44, 125.72, 123.70, 118.64, 115.76, 111.42, 67.14 (2C), 64.66, 56.20, 45.64 (2C), 30.22 (2C), 27.63 (2C), 27.19. LC-MS: *t*_R_ = 0.590 min (Purity = 98%); *m/z* = 462.2 [M+H]^+^ (anal. calcd. for C_26_H_31_N_5_O_3_: *m/z* = 461.2).

**2.2. *In vitro* asexual blood stage (ABS) antiplasmodial activity**

*In vitro* 72 h parasite lactate dehydrogenase (pLDH) antiplasmodial assays were carried out as previously described.^[2]^ For the *in vitro* 96 h SYBR Green I-based antiplasmodial assays used for the isobologram studies, the asexual *Pf*NF54 parasite strain was obtained from the Malaria Research and Reference Reagent Resource Centre (MR4 BEI Resources, Manassas, USA) and cultivated under adapted conditions as previously described.^[3]^ Parasites were maintained daily at 5% haematocrit (A^+^/O^+^ human erythrocytes) in complete culture media (RPMI 1640 supplemented with 25 mM HEPES, 20 mM d-glucose, 200 μM hypoxanthine, 0.2% (w/v) sodium bicarbonate (Sigma-Aldrich), 24 μg/mL gentamicin, and 0.5% (w/v) AlbuMAX II, Gibco) under hypoxic conditions (90% N_2_, 5% O_2_ and 5% CO_2_, AFROX, SA) at 37°C with agitation. Cultures were synchronised to 95% ring stage using 5% (w/v) d-sorbitol (Sigma-Aldrich). Parasite growth and morphology were monitored microscopically using Giemsa-stained thin smears at 1 000× magnification. The SYBR Green I parasite proliferation assay was performed with *Pf*NF54 parasites (1% parasitaemia, 1% haematocrit) for 96 h under hypoxic conditions, using chloroquine as an internal reference control. Following incubation, parasite proliferation was determined after addition of a 1× SYBR Green I lysis buffer (0.2% μL/mL of 10 000× SYBR Green I, 20 mM Tris-HCl, pH 7.5, 5 mM EDTA, 0.008% (w/v) saponin and 0.08% Triton X-100) and incubated for 1 h at room temperature in the dark. Fluorescence was quantified with the GloMaxR-Multi+ Detection System (485 nm/538 nm). *In vitro* IC_50_ values were determined using GraphPad Prism V6 (log(inhibitor) vs normalised response – variable slope). Data represent three independent biological repeats (*n* = 3), each performed in technical triplicates.

**2.3. *In vitro* gametocytocidal assays**

Stage-specific gametocytocidal action of AZD0156 was determined using against immature (> 90% stage II–III), late-stage (> 90% stage IV–V) and mature (> 95% stage V) gametocytes using the *P. falciparum* luciferase reporter line, 3D7*elo*1-*pfs16*-CBG99 (kind gift from Pietro Alano, ISS, Italy), as previously described.^[4]^ Drug assays were set up at 1.5% gametocytaemia and 2% haematocrit for a 48 h incubation under drug pressure under the same hypoxic conditions as above at 37°C, with methylene blue and MMV390048 serving as internal reference controls. The luciferase reporter assay was performed using 0.5 mM non-lysing d-luciferin (Regis Technologies) in citrate buffer (50 mM citric acid, 50 mM trisodium citrate hydrate) with bioluminescence detection using the GloMax^®^-Multi Detection System with Instinct^®^ software. *In vitro* IC_50_ values were determined using GraphPad Prism V8 software (as described above) for three independent biological repeats (*n* = 3) each performed in technical duplicates.

**2.4. Male gamete assays**

The male gamete exflagellation inhibition assay^[5]^ was performed on *Pf*NF54 mature (> 95% stage V) gametocytes treated with 2 µM compound under hypoxic conditions at 37°C (2 µM methylene blue as positive control). Male gametogenesis was induced in ookinete medium (containing 100 µM xanthurenic acid (Sigma-Aldrich) and 50% A^+^ male serum) with 100 µM xanthurenic acid (Sigma-Aldrich) at room temperature for 16 min in a Neubauer chamber followed by capturing of temporal movement of exflagellating centres by video microscopy (Carl Zeiss NT 6V/10 W Stab microscope, MicroCapture camera, 10× magnification) for 8 min at 30-second intervals for 8–10 sec. Total exflagellating centres were quantified using ICY (open-source imaging software GPLv3) normalised to an untreated control.

**2.5. Standard membrane feeding assay (SMFA)**

Performed as previously described.^[6]^ Briefly, the SMFA was carried out using *Anopheles coluzzii s.s.* females (colonised in 1975 from The Gambia, G3 colony).^[7]^ The mosquitoes were maintained under BSL2 insectary conditions (80% humidity, 25°C, 12 h day/12 h night cycle with 50 min dusk/dawn transitions) with *ad libitum* access to 10% sucrose supplemented with 0.05% (v/v) 4-aminobenzoic acid. Mature *Pf*NF54 gametocytes (> 98% stage V, 1.5 to 2.5% gametocytaemia, and 50% haematocrit) were treated with 2 μM AZD0156 with DMSO as a vehicle control for 48 h before mosquito feeding. Two independent biological experiments were carried out (total of 25 mosquitoes in treated control groups). The TRA (transmission-reducing activity, reduction in oocyst intensity: % TRA $\frac{Ci-Ti}{Ci} 100$, where *i*: oocyst number (intensity), *C*: control and *T*: treated) and the TBA, which measures the reduction in prevalence of mosquitoes infected with oocysts (percentage of block of transmission or reduction in prevalence, % TBA: $\frac{Cp-Tp}{Cp} 100$, where *p*: oocyst prevalence (intensity), *C*: control and *T*: treated^[8]^) were determined. Data were analysed using GraphPad Prism, and the Mann-Whitney U test was used to compare the statistical significance between the treatment and control groups.

**2.6. Resistance selection**

Selection of parasites resistant to AZD0156 was carried out as previously described.^[9]^ Duplicate flasks containing 100 mL of 10^9^ *Pf*Dd2 (clone B2) ABS parasites starting at 3% parasitaemia were cultured under constant drug pressure. Media containing 3× IC_50_ of ADZ0156 (360 nM) was refreshed daily for the first seven days, while cultures were monitored to confirm clearance of sensitive parasites. Fresh uninfected RBCs (0.5% haematocrit) were added on day 7 and from day 14, the culture volume was reduced by 50% each week in drug-free media. When recrudescence was observed, resistant parasites were maintained under drug pressure at a volume of 50 mL until a high parasitaemia (> 5%) was achieved. Aliquots of the bulk resistant cultures were frozen in cryovials before cloning by limiting dilution in 96-well plates (seeded at 0.25 or 0.5 parasites/well).^[10]^ After three weeks of cloning, positive wells were detected using the pLDH assay detection protocol. Four individual clones were expanded and profiled, but only two (D6 and F3) were harvested by saponin lysis at the trophozoite stage and genomic DNA was extracted using the Qiagen QIAamp DNA Blood Midi Kit.

Whole-genome sequencing was performed using a Nextera Flex DNA library kit and multiplexed on a MiSeq flow cell to generate 300 bp paired-end reads. Sequences were aligned to the *Pf*3D7 reference genome (PlasmoDB-48_Pfalciparum3D7; https://plasmodb.org/plasmo/app/downloads/release-48/Pfalciparum3D7/fasta/) using the Burrow-Wheeler Alignment (BWA version 0.7.17). PCR duplicates and unmapped reads were filtered out using Samtools (version 1.13) and Picard MarkDuplicates (GATK version 4.2.2). Base quality scores were recalibrated using GATK BaseRecalibrator (GATK version 4.2.2). GATK HaplotypeCaller (GATK version 4.2.2) was used to identify all possible single nucleotide variants (SNVs) in test parasite lines filtered based on quality scores (variant quality as function of depth QD > 1.5, mapping quality > 40, min base quality score > 18, read depth > 5) to obtain high-quality single nucleotide polymorphisms (SNPs) that were annotated using SnpEff version 4.3t.^[11]^ Comparative SNP analyses between the clones and the control *Pf*Dd2 line were performed to generate the final list of SNPs (Figure S1A). BIC-Seq version 1.1.2^[12]^ was used to discover copy number variants (CNVs) against the parental strain using the Bayesian statistical model. SNPs and CNVs were visually inspected and confirmed using Integrative Genome Viewer (IGV). All gene annotations in the analysis were based on PlasmoDB-48_Pfalciparum3D7 (https://plasmodb.org/plasmo/app/downloads/release-48/Pfalciparum3D7/gff/).

**2.7. Cross-resistance studies**

The [^3^H]-hypoxanthine incorporation assay was used to assess cross-resistance as previously described.^[13]^ Briefly, a culture of *Pf*3D7A strain parasitised red blood cells (0.5% parasitaemia and 2% haematocrit in RPMI-1640 supplemented with 5 g/L AlbuMAX and 5 µM hypoxanthine) was exposed to 3-fold serial dilutions of the compound. Plates were incubated for 24 h at 37ºC (in 5% CO_2_, 5% O_2_, 90% N_2_). After the 24 h incubation, ^3^H-hypoxanthine was added, and plates were incubated for 24 h more before being frozen at -70ºC. Parasites were harvested on a glass fibre filter using a TOMTEC Cell Harvester 96. Filters were dried, and melt-on scintillator sheets were used to determine the incorporation of [^3^H]-hypoxanthine measuring radioactivity in a MicroBeta B-counter WALLAC. IC_50_ data was analysed using Microsoft Excel XLfit. Raw data were normalised using the positive control (parasitised red blood cells with DMSO) and the negative control (parasitised red blood cells exposed to artesunate 2 µM).

**2.8. *In vitro* *Pv*PI4Kβ inhibition assays**

Full-length *Pv*PI4Kβ (PVX_098050) recombinant protein was expressed in a baculovirus-insect cell expression system and purified as previously described.^[14]^ Briefly, N-terminal His-tagged recombinant *Pv*PI4Kβ protein was purified using a HisTrap HP column (GE Healthcare), followed by size-exclusion chromatography (HiLoad 16/600 Superdex 200 pg column, GE Healthcare).

*Pv*PI4Kβ kinase inhibition assays were performed using the ADP-Glo Kinase Assay kit (Promega) to measure ADP formation. Briefly, a three-fold serial dilution of each inhibitor was carried out in DMSO and inhibitors were subsequently diluted into assay buffer (25 mM HEPES pH 7.4, 100 mM NaCl, 3 mM MgCl_2_, 1 mM DTT, 0.025 mg/mL BSA, 0.2% (v/v) Triton X-100) to 1.5× the final concentration. 2 µL of each inhibitor dilution was transferred into a white 384 shallow-well plate (Nunc #264706). A MANTIS^®^ Liquid Handler (Formulatrix) was used to dispense the remaining assay components. 0.5 µL *Pv*PI4Kβ protein was added and following a five-minute pre-incubation with inhibitor, 0.5 µL substrate buffer (ATP and PI) was added to each well. The final 3 µL kinase reaction contains ~6 nM *Pv*PI4Kβ protein, 10 µM ATP, 0.1 mg/mL PI, 1% (v/v) DMSO and inhibitor in assay buffer. Reactions were incubated for 40 min at 22°C (resulting in < 10% ATP conversion). ADP formation was measured using the ADP-Glo Kinase Kit (Promega). Briefly, 2 µL ADP-Glo reagent containing 10 mM MgCl_2_ was added to each well and incubated for 40 min at 22°C to deplete the remaining ATP. 2 µL of Kinase Detection Reagent was then added and the reaction was incubated for a further 30 min at 22°C. The plate was sealed with an adhesive foil seal for all incubation steps. Luminescent signal was measured using the EnSpire Multimode Plate Reader (PerkinElmer). The data was normalised based on the 100% activity controls (1% DMSO only) and the 100% inhibition controls (10 µM sapanisertib^[2]^). The mean IC_50_ value was calculated from *n* ≥ 3 independent experiments, each with technical duplicates (log(inhibitor) vs. normalised response – variable slope). IC_50_ values within three-fold from independent experiments are considered reproducible.

Note: The *Pv*PI4K*K_m_*^ATP^ in the ADP-Glo assay is 300 μM (unpublished data). Therefore, the assay is carried out at an ATP concentration well below *K_m._* Under these conditions, the IC_50_ approximates *K*_i_ although it should be noted that the potency of AZD0156 is near the lower limit of the assay based on the enzyme concentration so the potency may be underestimated due to tight binding assay conditions. Cellular ATP concentrations are typically in the low millimolar range, providing an explanation for the difference between enzyme and whole-cell activity. IC_50_ values at cellular ATP concentrations can be calculated using the Cheng-Prusoff Equation. For example, the calculated *Pv*PI4K IC_50_ at 3 mM ATP ~ 55 nM.

**2.9. Conditional knockdown studies**

Synchronous ring-stage PI4Kβ (PF3D7_0509800) cKD parasites, as well as a control parasite line expressing an aptamer-regulatable fluorescent protein were maintained in the presence of high aTc (500 nM) or no aTc and distributed into 384-well polystyrene microplates. Stock solutions of compounds were serially diluted and transferred to the parasite-containing plates. DMSO and dihydroartemisinin treatment (500 nM) served as reference controls. Luminescence was measured after 72 h using the Renilla-Glo Luciferase Assay System (Promega E2750) and the GloMax Discover Multimode Microplate Reader (Promega), and IC_50_ values were obtained from corrected dose-response curves using GraphPad Prism.

**2.10. Profiling against off-target kinases *Hs*PI4Kβ, *Hs*MAP4K4 and *Hs*MINK1**

The biochemical inhibitory activities (IC_50_ values) of AZD0156 against the human kinases *Hs*PI4Kβ, *Hs*MAP4K4 and *Hs*MINK1 were measured at Reaction Biology with an ADP-Glo Kinase Assay platform (Promega). AZD0156 was tested in 10-dose IC_50_ mode with a three-fold serial dilution starting at 10 μM. Control compound PIK-93 was tested in 10-dose IC_50_ mode with three-fold serial dilution starting at 10 μM while control compound staurosporine was tested in 10-dose IC_50_ mode with four-fold serial dilution starting at 20 μM. Reactions were carried out at 10 μM ATP. A detailed description of the ADP-Glo assay is available online (https://www.promega.co.uk/products/cell-signaling/kinaseassays-and-kinase-biology/adp-glo-kinase-assay/?catNum=V6930).

**2.11. *In vitro* *Pf*PKG inhibition assays**

Full-length *Pf*PKG (PF3D7_1436600) was expressed in *E. coli* Rosetta 2 (Novagen, EMD_BIO-71402) as previously described, with *Pf*PKG IC_50_ assays also performed on the basis of previously described methods using the ADP-Glo Kinase Assay (Promega) to measure adenosine diphosphate (ADP) formation.^[2]^ Briefly, the N-terminal His-tagged recombinant *Pf*PKG protein was purified using a HisTrap HP column (GE Healthcare), followed by anion exchange and size exclusion chromatography (HiLoad 16/600 Superdex 200-pg column, GE Healthcare). Final buffer composition of purified protein was 50 mM tris-HCl (pH 8.0), 150 mM NaCl, 10 mM β-mercaptoethanol and 10% glycerol.

Inhibitor IC_50_ assays were performed using the ADP-Glo Kinase Assay to measure ADP formation. Serially-diluted inhibitors were mixed with *Pf*PKG protein, substrate buffer (ATP and peptide GRTGRRNSI-NH_2_) and assay buffer in a 384-well plate. Reactions were incubated for 45 min at 22°C, followed by sequential additions of ADP-Glo reagent and kinase detection reagent, with further incubations to deplete ATP and generate luminescence. Luminescence was measured on an EnSpire Multimode Plate Reader (PerkinElmer). The data were normalised on the basis of the 100% activity controls (1% DMSO only) and the 100% inhibition controls (10 μM *Pf*PKG inhibitor ML10, LifeArc). Mean IC_50_ values were calculated from *n* ≥ 3 independent experiments, each with technical duplicates [log(inhibitor) versus normalised response − variable slope].

**2.12. NP40-mediated β-haematin inhibition assay**

The β-haematin inhibition assay was followed according to the method outlined by Carter et al.^[15]^ Test compounds were prepared in DMSO (20 mM) and serially diluted in a 96-well plate. Haematin (25 mM) in acetate buffer (pH 4.8) was added to wells and incubated at 37°C for 5 hours. β-haematin inhibition was analysed using the pyridine-ferrichrome method^[16]^, with absorbance measured at 405 nm on a SpectraMax P340 plate reader. Sigmoidal dose-response curves were generated using GraphPad Prism V7 software.

**2.13. Metabolomics**

In vitro *cultivation of* P. falciparum *for treatments and extractions*

*Pf*3D7 parasite lines were cultured at 2% haematocrit under standard growth conditions.^[17]^ Barring the cycle before metabolite extraction, parasites were synchronised twice two days before extraction by sorbitol lysis^[18]^ and grown to the trophozoite stage at ~10% parasitaemia. Infected red blood cells (iRBCs) were magnetically purified using MACS CS columns and VarioMacs magnets.^[19]^ Metabolite extracts were prepared from purified iRBCs as described previously,^[20]^ resulting in a 1 mL extraction in 90% MeOH spiked with 0.5 µM [^13^C4,^15^N1]-labelled aspartate standard. Extracts were vortexed and centrifuged (Eppendorf Centrifuge 5424R and FA-45-24-11 Rotor) for 10 min at 4°C at 15 000 rpm. Supernatant was transferred into 1.5 mL tubes, dried under nitrogen, and stored at -80°C.

*Metabolomic profiling of drug-treated parasites using mass spectrometry*

Extracts were thawed on ice and resuspended in ice-cold HPLC-grade water containing 1 µM chlorpropamide to a concentration of 1 x 10^6^ cells/µL. Extracted samples were vortexed and centrifuged for 10 min at 4°C at 15 000 rpm. The supernatant was added to 800 µL CRIMP vials for mass spectrometry loading. Samples were separated using an XSelect HSS T3 2.5 µm C18 Waters column with a 25-minute gradient of 3% aqueous methanol, 15 mM acetic acid, and 10 mM tributylamine and were run in negative ionisation mode on a Thermo Exactive Plus orbitrap.^[21]^

Mass spectrometry data were imported into the el-MAVEN software package for peak picking^[22]^ and were normalised using the [^13^C4,^15^N1] aspartate standard. Fold change was calculated by and uploaded to Rstudio (http://www.rstudio.com/) using packages Hyperspec and Suprahex.^[23]^ Metabolic profiles (log2 fold changes) were transferred to a hexagonal map with 113 metabolites.^[20]^ All metabolomics data are available publicly through the NCBI Metabolomics Workbench (Project ID: PR002010).

**2.14. Isobologram analysis**

Fixed-ratio isobologram analysis of AZD0156 with MMV390048 and dihydroartemisinin (DHA) against ABS and late-stage gametocytes was performed as previously described^[24]^, with DHA and MMV390048 combinations as reference. Parasites were treated with fixed ratios (5:0, 4:1, 3:2, 2:3, 1:4 and 0:5) for AZD0156/DHA with MMV390048 using the SYBR Green I proliferative assay for ABS and the d-luciferin assay for late-stage gametocytes (described above) as readouts to determine the fractional inhibitory concentrations (FIC) for the respective combinations. Isobolograms were obtained by plotting paired FIC linearly, using the average of three biological replicates (unless otherwise stated), each performed in technical duplicates (ABS) or triplicates (late-stage gametocytes) paired FIC values for each of the drugs in combination, and the mean FIC values (ΣFIC) were determined to delineate synergism (< 0.8), additivity/indifference (0.8–1.4) or antagonism (> 1.4).^[5]^

**2.15. *In vitro* parasite reduction ratio (PRR) assays**

The *in vitro* PRR assays were carried out as previously described.^[25]^ The parasite killing profile is estimated by culturing unlabelled erythrocytes infected with the 3D7 strain in the presence of the tested compound at a concentration corresponding to 10× IC_50_. Parasites were drug-treated for 24 h and 48 h. Tested compounds were renewed after the first 24 h of treatment by removing old media and replenishing with new culture media with fresh drug. After treatment, the compound was removed and the culture diluted 1/3rd using fresh erythrocytes (2% haematocrit) previously labelled with CFDA-SE (carboxylfluorescein diacetate succinimidyl ester, Life Technologies) as described. Following a further 48 h incubation, the ability of treated parasites to establish new infections in fresh, labelled erythrocytes was detected by quantification of double stained erythrocytes using two-colour flow cytometry (Attune NxT Flow Cytometer, ThermoFisher) after labelling of parasite DNA with Hoechst 33342 (Sigma). The Hoechst 33342 was excited by a laser at 405 nm and detected by a 440/50 nm filter (VL1). CFDA-SE was excited by a blue laser 488 nm and detected by a 530/30 nm filter. Samples were analysed using the Attune NxT software package. Parasite viability was shown as the percentage of infected CFDA-SE-stained erythrocytes in drug-treated samples at 24 h or 48 h, using labelled erythrocytes and labelling of parasite DNA from untreated cultures as a control. Chloroquine, pyrimethamine, atovaquone and artesunate were used in each assay to validate the assay and allow for comparative classification of the killing rate of the tested compound. Comparative data for MMV390048 were taken from Paquet et al.^[26]^

**2.16. Rate- and stage-specific morphological evaluations**

*In vitro* cultured parasites (ABS and GC) were treated with AZD0156 and MMV390048 at IC_90_ with the rate of activity and stage-specific killing monitored morphologically using Giemsa-stained thin smears. Asexual blood stage parasites were treated at ring, early/late trophozoite and schizont stages, sampling every 12 h over a 48 h time period, with stage IV and V gametocytes being treated and monitored over 24 h and 48 h. Images were acquired using a Nikon Eclipse 50i microscope at 100× oil immersion magnification with NIS Elements software and processed through a set-scale with Fiji, significance was determined using a Mann-Whitney U test. Late-stage gametocytes (> 90% IV–V) treated with MMV390048 (IC_90_ for 24 h), were stained with 30 nM MitoTracker Red CMXRos (Invitrogen by Thermo Scientific, USA) for 10 min at 37°C. Following treatment, gametocytes were washed (2–3 times) and resuspended in pre-warmed sterile filtered Ringer’s solution (pH 7.4; 25 mM HEPES; 122.5 mM NaCl; 1 mM NaH_2_PO_4_; 5.4 mM KCl; 11 mM d-glucose; 1.2 mM CaCl_2_; 0.8 mM MgCl_2_) and visualised using a Zeiss Axio Fluorescence Microscope and the Zeiss ZEN Blue software (Zeiss, Germany).

**2.17. *In vivo* pharmacokinetic studies, cytotoxicity and cardiotoxicity**

As previously described.^[27]^ For cytotoxicity measurements against the HepG2 cell line, human Caucasian hepatocellular carcinoma cells (HepG2, Merck) cultivated *in vitro* (DMEM media supplemented with 10% (v/v) heat-inactivated foetal bovine serum and 1% (v/v) penicillin/streptomycin (Hyclone, USA)). Trypsinised (0.25% Trypsin-EDTA, Sigma-Aldrich) cells (10^5^ cells/well) were treated with 2 μM AZD0156 for 24 h at 37°C under 5% CO_2_ and 95% humidity. Cytotoxicity was determined using the lactate dehydrogenase release assay (CytoSelect Inc.) as per manufacturer’s instructions. Data represent a single biological repeat performed in technical triplicates (mean ± SD).

**2.18. *In vivo* efficacy study**

Tests against *P. berghei* were conducted in mice following oral administration of 50 mg/kg/day as described previously.^[27]^

**Ethics**

Parasitology studies based at the University of Cape Town (pLDH assays, PRR experiments and resistance selections) were covered under ethical approval from the Faculty of Health Sciences Human Research Ethics Committee (HREC 890/2019, HREC 645/2023 and AEC020-010).

All pharmacokinetic (PK) studies and procedures were conducted with prior approval of the animal ethics committee of the University of Cape Town (AEC022_004) in accordance with the South African National Standard (SANS 10386:008) for the Care and Use of Animals for Scientific Purposes^[28]^ and guidelines from the Department of Health^[29]^.

Parasitology work (transmission-blocking studies) and consent-based volunteer human blood donation (University of Pretoria) were covered under ethical approval from the University of Pretoria Health Sciences Ethics Committee (506/2018) and Natural and Agricultural Sciences Ethics Committee (180000094). Standard membrane feeding assays were covered under ethical approval University of the Witwatersrand Human Research Ethics Committee (M130569) and Animal Ethics Committee (20190701-7O).

The human biological samples used in the parasitology studies carried out at GSK (cross-resistance studies and PRR assays) were sourced ethically and their research use was in accord with the terms of the informed consents under an IRB/EC approved protocol.

Animal experiments against *P. berghei* were carried out at the Swiss Tropical and Public Health Institute and adhered to local and national regulations of laboratory animal welfare in Switzerland (awarded permission number BS1731/BL519 and BL521). Protocols are regularly reviewed and revised following approval by the local authority (Veterinäramt Basel-Stadt and Basel-Land).

**3. COMPUTATIONAL METHODS**

**3.1. *In silico* molecular docking**

The *Pf*PI4Kβ homology model^[30]^ was prepared for docking using the Maestro protein preparation tool (Schrodinger Release 2021-3: Maestro, Schrodinger, LLC, New York, NY, 2021). Once prepared, a docking grid was created around the binding site using the GLIDE docking grid generation tool with a hydrogen bonding constraint set on the backbone amide of the critical hinge valine (*Pf*PI4Kβ, V1357). The structure of AZD0156 was then drawn using the Maestro 2D sketcher, after which it was prepared using the LigPrep tool at pH 7.0. AZD0156 was then docked using GLIDE at standard precision with the hydrogen bond constraint implemented.^[31]^ Full residues within a 5.0 Å radius of the ligand were minimised using PRIME minimisation in the function and the OPLS4 force field. Computational figures were generated using PyMol.^[32]^

**REFERENCES**

[1] K. G. Pike, B. Barlaam, E. Cadogan, A. Campbell, Y. Chen, N. Colclough, N. L. Davies, C. de-Almeida, S. L. Degorce, M. Didelot, A. Dishington, R. Ducray, S. T. Durant, L. A. Hassall, J. Holmes, G. D. Hughes, P. A. MacFaul, K. R. Mulholland, T. M. McGuire, G. Ouvry, M. Pass, G. Robb, N. Stratton, Z. Wang, J. Wilson, B. Zhai, K. Zhao, N. Al-Huniti, *J Med Chem* **2018**, *61*, 3823-3841.

[2] L. B. Arendse, J. M. Murithi, T. Qahash, C. F. A. Pasaje, L. C. Godoy, S. Dey, L. Gibhard, S. Ghidelli-Disse, G. Drewes, M. Bantscheff, M. J. Lafuente-Monasterio, S. Fienberg, L. Wambua, S. Gachuhi, D. Coertzen, M. van der Watt, J. Reader, A. S. Aswat, E. Erlank, N. Venter, N. Mittal, M. R. Luth, S. Ottilie, E. A. Winzeler, L. L. Koekemoer, L. M. Birkholtz, J. C. Niles, M. Llinas, D. A. Fidock, K. Chibale, *Sci Transl Med* **2022**, *14*, eabo7219.

[3] M. Leshabane, G. A. Dziwornu, D. Coertzen, J. Reader, P. Moyo, M. van der Watt, K. Chisanga, C. Nsanzubuhoro, R. Ferger, E. Erlank, N. Venter, L. Koekemoer, K. Chibale, L. M. Birkholtz, *ACS Infect Dis* **2021**, *7*, 1945-1955.

[4] L. Cevenini, G. Camarda, E. Michelini, G. Siciliano, M. M. Calabretta, R. Bona, T. R. Kumar, A. Cara, B. R. Branchini, D. A. Fidock, A. Roda, P. Alano, *Anal Chem* **2014**, *86*, 8814-8821.

[5] J. Reader, M. E. van der Watt, D. Taylor, C. Le Manach, N. Mittal, S. Ottilie, A. Theron, P. Moyo, E. Erlank, L. Nardini, N. Venter, S. Lauterbach, B. Bezuidenhout, A. Horatscheck, A. van Heerden, N. J. Spillman, A. N. Cowell, J. Connacher, D. Opperman, L. M. Orchard, M. Llinas, E. S. Istvan, D. E. Goldberg, G. A. Boyle, D. Calvo, D. Mancama, T. L. Coetzer, E. A. Winzeler, J. Duffy, L. L. Koekemoer, G. Basarab, K. Chibale, L. M. Birkholtz, *Nat Commun* **2021**, *12*, 269.

[6] R. H. Hunt, B. D. Brooke, C. Pillay, L. L. Koekemoer, M. Coetzee, *Med Vet Entomol* **2005**, *19*, 271-275.

[7] (a) M. Coetzee, R. H. Hunt, R. Wilkerson, A. Della Torre, M. B. Coulibaly, N. J. Besansky, *Zootaxa* **2013**, *3619*, 246-274; (b) C. Fanello, F. Santolamazza, A. della Torre, *Med Vet Entomol* **2002**, *16*, 461-464.

[8] K. Miura, B. J. Swihart, B. Deng, L. Zhou, T. P. Pham, A. Diouf, T. Burton, M. P. Fay, C. A. Long, *Vaccine* **2016**, *34*, 4145-4151.

[9] D. Dorjsuren, R. T. Eastman, K. J. Wicht, D. Jansen, D. C. Talley, B. A. Sigmon, A. V. Zakharov, N. Roncal, A. T. Girvin, Y. Antonova-Koch, P. M. Will, P. Shah, H. Sun, C. Klumpp-Thomas, S. Mok, T. Yeo, S. Meister, J. J. Marugan, L. S. Ross, X. Xu, D. J. Maloney, A. Jadhav, B. T. Mott, R. J. Sciotti, E. A. Winzeler, N. C. Waters, R. F. Campbell, W. Huang, A. Simeonov, D. A. Fidock, *Sci Rep* **2021**, *11*, 2121.

[10] I. D. Goodyer, T. F. Taraschi, *Exp Parasitol* **1997**, *86*, 158-160.

[11] P. Cingolani, A. Platts, L. Wang le, M. Coon, T. Nguyen, L. Wang, S. J. Land, X. Lu, D. M. Ruden, *Fly (Austin)* **2012**, *6*, 80-92.

[12] R. Xi, J. Luquette, A. Hadjipanayis, T.-M. Kim, P. J. Park, *Genome Biology* **2010**, *11*.

[13] C. de Cozar, I. Caballero, G. Colmenarejo, L. M. Sanz, E. Alvarez-Ruiz, F. J. Gamo, C. Cid, *Antimicrob Agents Chemother* **2016**, *60*, 5949-5956.

[14] (a) P. M. Cheuka, L. Centani, L. B. Arendse, S. Fienberg, L. Wambua, S. S. Renga, G. A. Dziwornu, M. Kumar, N. Lawrence, D. Taylor, S. Wittlin, D. Coertzen, J. Reader, M. van der Watt, L.-M. Birkholtz, K. Chibale, *ACS Infectious Diseases* **2021**, *7*, 34-46; (b) C. W. McNamara, M. C. S. Lee, C. S. Lim, S. H. Lim, J. Roland, A. Nagle, O. Simon, B. K. S. Yeung, A. K. Chatterjee, S. L. McCormack, M. J. Manary, A.-M. Zeeman, K. J. Dechering, T. R. S. Kumar, P. P. Henrich, K. Gagaring, M. Ibanez, N. Kato, K. L. Kuhen, C. Fischli, M. Rottmann, D. M. Plouffe, B. Bursulaya, S. Meister, L. Rameh, J. Trappe, D. Haasen, M. Timmerman, R. W. Sauerwein, R. Suwanarusk, B. Russell, L. Renia, F. Nosten, D. C. Tully, C. H. M. Kocken, R. J. Glynne, C. Bodenreider, D. A. Fidock, T. T. Diagana, E. A. Winzeler, *Nature* **2013**, *504*, 248-253.

[15] M. D. Carter, V. V. Phelan, R. D. Sandlin, B. O. Bachmann, D. W. Wright, *Comb Chem High Throughput Screen* **2010**, *13*, 285-292.

[16] K. K. Ncokazi, T. J. Egan, *Anal Biochem* **2005**, *338*, 306-319.

[17] W. Trager, J. B. Jensen, *Science* **1976**, *193*, 673-675.

[18] C. Lambros, J. P. Vanderberg, *J Parasitol* **1979**, *65*, 418-420.

[19] C. C. Kim, E. B. Wilson, J. L. DeRisi, *Malar J* **2010**, *9*, 17.

[20] E. L. Allman, H. J. Painter, J. Samra, M. Carrasquilla, M. Llinas, *Antimicrob Agents Chemother* **2016**, *60*, 6635-6649.

[21] Lu, *Anal Chem* **2010**.

[22] S. Agrawal, S. Kumar, R. Sehgal, S. George, R. Gupta, S. Poddar, A. Jha, S. Pathak, *Methods Mol Biol* **2019**, *1978*, 301-321.

[23] H. Fang, J. Gough, *Biochem Biophys Res Commun* **2014**, *443*, 285-289.

[24] (a) D. A. van Schalkwyk, M. K. Riscoe, S. Pou, R. W. Winter, A. Nilsen, M. Duffey, R. W. Moon, C. J. Sutherland, *Antimicrob Agents Chemother* **2020**, *64*; (b) Q. L. Fivelman, I. S. Adagu, D. C. Warhurst, *Antimicrob Agents Chemother* **2004**, *48*, 4097-4102; (c) D. Coertzen, J. Reader, M. van der Watt, S. H. Nondaba, L. Gibhard, L. Wiesner, P. Smith, S. D'Alessandro, D. Taramelli, H. N. Wong, J. L. du Preez, R. W. K. Wu, L. M. Birkholtz, R. K. Haynes, *Antimicrob Agents Chemother* **2018**, *62*; (d) D. A. van Schalkwyk, W. Priebe, K. J. Saliba, *J Pharmacol Exp Ther* **2008**, *327*, 511-517.

[25] M. Linares, S. Viera, B. Crespo, V. Franco, M. G. Gomez-Lorenzo, M. B. Jimenez-Diaz, I. Angulo-Barturen, L. M. Sanz, F. J. Gamo, *Malar J* **2015**, *14*, 441.

[26] T. Paquet, C. Le Manach, D. G. Cabrera, Y. Younis, P. P. Henrich, T. S. Abraham, M. C. S. Lee, R. Basak, S. Ghidelli-Disse, M. J. Lafuente-Monasterio, M. Bantscheff, A. Ruecker, A. M. Blagborough, S. E. Zakutansky, A. M. Zeeman, K. L. White, D. M. Shackleford, J. Mannila, J. Morizzi, C. Scheurer, I. Angulo-Barturen, M. S. Martinez, S. Ferrer, L. M. Sanz, F. J. Gamo, J. Reader, M. Botha, K. J. Dechering, R. W. Sauerwein, A. Tungtaeng, P. Vanachayangkul, C. S. Lim, J. Burrows, M. J. Witty, K. C. Marsh, C. Bodenreider, R. Rochford, S. M. Solapure, M. B. Jimenez-Diaz, S. Wittlin, S. A. Charman, C. Donini, B. Campo, L. M. Birkholtz, K. K. Hanson, G. Drewes, C. H. M. Kocken, M. J. Delves, D. Leroy, D. A. Fidock, D. Waterson, L. J. Street, K. Chibale, *Sci Transl Med* **2017**, *9*.

[27] C. Brunschwig, N. Lawrence, D. Taylor, E. Abay, M. Njoroge, G. S. Basarab, C. Le Manach, T. Paquet, D. G. Cabrera, A. T. Nchinda, C. de Kock, L. Wiesner, P. Denti, D. Waterson, B. Blasco, D. Leroy, M. J. Witty, C. Donini, J. Duffy, S. Wittlin, K. L. White, S. A. Charman, M. B. Jimenez-Diaz, I. Angulo-Barturen, E. Herreros, F. J. Gamo, R. Rochford, D. Mancama, T. L. Coetzer, M. E. van der Watt, J. Reader, L. M. Birkholtz, K. C. Marsh, S. M. Solapure, J. E. Burke, J. A. McPhail, M. Vanaerschot, D. A. Fidock, P. V. Fish, P. Siegl, D. A. Smith, G. Wirjanata, R. Noviyanti, R. N. Price, J. Marfurt, K. D. Silue, L. J. Street, K. Chibale, *Antimicrob Agents Chemother* **2018**, *62*.

[28] South African Bureau of Standards, *South African National Standard: The care and use of animals for scientific purposes*, 1 ed., SABS Standards Division, Pretoria, South Africa, **2008**.

[29] Department of Health, *Ethics in health research: Principles, processes and structures*, 2 ed., Department of Health, Republic of South Africa, Pretoria, South Africa, **2015**.

[30] S. Fienberg, C. J. Eyermann, L. B. Arendse, G. S. Basarab, J. A. McPhail, J. E. Burke, K. Chibale, *ACS Infect Dis* **2020**, *6*, 3048-3063.

[31] R. A. Friesner, J. L. Banks, R. B. Murphy, T. A. Halgren, J. J. Klicic, D. T. Mainz, M. P. Repasky, E. H. Knoll, M. Shelley, J. K. Perry, D. E. Shaw, P. Francis, P. S. Shenkin, *J Med Chem* **2004**, *47*, 1739-1749.

[32] Schrodinger, LLC, **2015**.
